# V_3_O_7_·H_2_O as a Cathode Material for Aqueous Mg^2+^/Na^+^ Hybrid Electrochemical Cells

**DOI:** 10.1021/acsomega.4c08983

**Published:** 2025-03-04

**Authors:** Daniela Söllinger, Julie Lam Chen, Jakub Zalesak, Jakob Praxmair, Simone Pokrant

**Affiliations:** Chemistry and Physics of Materials, University of Salzburg, Salzburg 5020, Austria

## Abstract

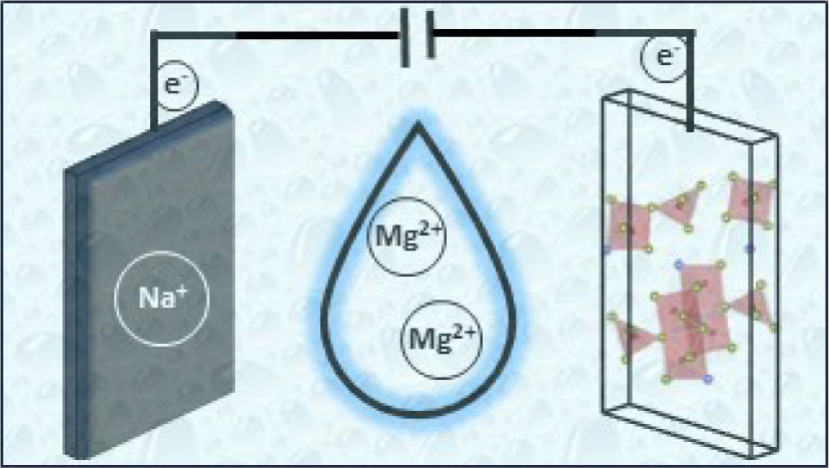

To improve safety and sustainability aspects of today’s energy storage solutions, aqueous post-lithium-ion electrochemical cells are promising concepts. In this context, we synthesize hydrated vanadium oxide (V_3_O_7_·H_2_O) via a hydrothermal process and investigate its electrochemical properties as a cathode in aqueous post-lithium-ion hybrid cells. This active material exhibits an excellent initial specific capacity of 404 mAh·g^–1^ at a practical current density of 100 mA·g^–1^ and a promising stability of 123 mAh·g^–1^ at a current density of 1 A·g^–1^ after 500 cycles upon Mg^2+^ and Na^+^ cointercalation. The insertion characteristics of this hybrid cell are investigated by cyclovoltammetry, operando XRD, and post-mortem transmission electron microscopy measurements. The results suggest that Na^+^ is inserted preferentially at lower potentials and that its effect fades quickly, while the Mg^2+^ contribution enables stable cycling, resulting in a competitive capacity retention of 73% after 500 cycles. In addition, we find that the charge transfer resistances are about one hundred times smaller in aqueous than in pure organic electrolyte as measured by electrochemical impedance spectroscopy. These results demonstrate that V_3_O_7_·H_2_O is a suitable cathode material for aqueous Mg^2+^/Na^+^ hybrid cells, enabling a performance that is comparable to cathodes in less sustainable and less safe organic electrolytes with respect to capacity and stability and is superior in transport-related properties.

## 1 Introduction

Aqueous electrolytes in batteries have the potential to prevent hazardous reactions inside an electrochemical cell since they are, in contrast to commercialized, organic electrolytes, not flammable.^1^ Furthermore, post-lithium-ion electrochemical cells such as Na-ion batteries (SIBs) or Mg-ion batteries (MIBs) are more sustainable compared to Li-ion batteries (LIBs) due to less environmentally harmful mining processes compared to Li and the higher abundance of Na and Mg in the Earth’s crust, resulting in improved accessibility worldwide.^2,3^ One of the challenges in postlithium ion batteries is related to the development of stable high-capacity cathodes. Table S1 summarizes representative cathode materials in aqueous Mg^2+^ and/or Na^+^ containing electrolytes, with a focus on Mg^2+^-containing electrolytes.^4,5^ Since Na and Mg metals react in contact with water, it is extremely challenging to use aqueous electrolytes in combination with the Na or Mg metal as a counter electrode. For this reason, many studies rely on activated carbon as a counter electrode.^6−9^ For example, Wang et al. investigated the magnesiation and demagnesiation of a Li_3_V_2_(PO_4_)_3_@C cathode material in an aqueous Mg^2+^-containing electrolyte.^10^ Using a 4 mol·kg^–1^ Mg(TFSI)_2_ electrolyte, activated carbon as a counter electrode, and Ag/AgCl as a reference electrodes, specific capacities of ∼115 mAh·g^–1^ were obtained at a current density of 100 mA·g^–1^. Besides the magnesiation and demagnesiation in Li_3_V_2_(PO_4_)_3_@C, Li^+^ was extracted during the initial cycle (IC) leading to the new host material LiV_2_(PO_4_)_3_. Although the amount of Li^+^ in the system was confirmed to be much smaller compared to Mg^2+^, it contributed at the beginning of the galvanostatic charge/discharge cycle resulting in enhanced performance.^10^ Studying the insertion of more than one active ion species in so-called hybrid batteries is an emerging field in battery research that has already led to enhanced electrochemical properties.^11−13^ In this context, the addition of binders containing potential insertion ions such as sodium carboxymethyl cellulose (Na-CMC) has resulted in improved electrochemical properties in various battery types because they enabled hybrid battery functionalities.^14,15^ Choi et al. reported that poly(acrylic acid) and Na-CMC were used as binders for Si-based anodes in LIBs, showing improved stabilities during electrochemical cycling compared to other binders such as PVDF.^14^ To further improve the electrochemical properties of aqueous lithium ion batteries by using the positive effect provided by hybrid cells, more research needs to be carried out on cathode materials, which allow the reversible inter- and deintercalation of various ions in aqueous electrolytes. One of these potential cathode materials is hydrated vanadium oxide V_3_O_7_·H_2_O (also written as H_2_V_3_O_8_, HVO). HVO has already shown promising electrochemical properties as a cathode material in several battery types due to its ability to intercalate various ions such as Li^+^,^16^ Na^+^,^17^ or Mg^2+^.^18^ Li et al. observed a specific capacity of 234 mAh·g^–1^ at a current density of 100 mA·g^–1^ in a LIB containing 5 M LiNO_3_ and 0.001 M LiOH aqueous electrolyte, with HVO as the cathode and a carbon electrode as the counter electrode.^19^ In a previous study, Söllinger et al. showed how small quantities of water influence the electrochemical properties of HVO for Mg^2+^ insertion in organic electrolyte.^20^ Since HVO-containing cathodes exhibit specific capacities of ∼300 mAh·g^–1^ in water-containing organic electrolytes with respect to Mg^2+^ intercalation, it is an interesting question of whether similar or even better electrochemical performance is reached in aqueous electrolyte. Furthermore, it has been shown that the structural changes of HVO during intercalation of Li^+^, Na^+^, Mg^2+^, or Zn^2+^ ions show similarities in single ion type cells.^21^ These results suggest that the coinsertion of several ion types in HVO is feasible. Therefore, the question arises if improved electrochemical properties can be reached in hybrid cell configurations, such as by the cointercalation of Na^+^ and Mg^2+^.

In this study, we will answer this question and add to the knowledge of hybrid cells using HVO as the cathode material for Mg^2+^ and Na^+^ insertion in an aqueous hybrid electrochemical cell. For this purpose, HVO is synthesized by a hydrothermal process. Its performance as a cathode material is assessed in a cell containing 0.5 M Mg(NO_3_)_2_ aqueous electrolyte. The activated carbon counter electrode contains Na-CMC as a binder to introduce small quantities of Na^+^ into the cell. The electrochemical performance of the established hybrid cell is investigated by galvanostatic cycling and cyclovoltammetry (CV). Furthermore, the crystal structure evolution of HVO during Mg^2+^ and Na^+^ ion insertion and extraction in aqueous electrolyte is investigated by operando XRD. To analyze the Mg^2+^ and Na^+^ distribution in HVO after the initial discharge, scanning transmission electron microscopy (STEM) and energy-dispersive X-ray spectroscopy (EDX) are carried out. Finally, the insertion process is studied by the galvanostatic intermittent titration technique (GITT) and electrochemical impedance spectroscopy (EIS) and is compared with the results obtained for HVO in a water-containing organic electrolyte (0.25 M Mg(ClO_4_)_2_·3.1H_2_O in acetonitrile).

## 2 Experimental Section

### 2.1 Synthesis

#### 2.1.1 V_3_O_7_·H_2_O (HVO)

The synthesis of V_3_O_7_·H_2_O is similar to previous studies and therefore described in more detail in the Supporting Information.^20,22,23^

#### 2.1.2 AC Pellets

The counter and reference electrode was prepared by mixing 2.8 g of AC and 0.6 g of carbon black in 20 mL THF with a vortex shaker for 10 min. In parallel, 0.24 g of Na-CMC was dissolved in 10 mL of deionized water, followed by the addition of 0.23 mL of SBR binder to the solution under stirring. In another vessel, 30 mg of ascorbic acid was dissolved in 5 mL of deionized water. Afterward, 0.28 g of graphite oxide was added to the solution, followed by ultrasonication for 5 min. All of the mixtures were added to the carbon compounds and further mixed with the vortex shaker for 5 min. Immediately afterward, 12–15 pellets were pressed with a diameter of around 15 mm and an average weight of 0.15 g. The pellets for operando XRD measurements had a diameter of 6 mm and an average weight of 0.09 g. All pellets were dried in a vacuum furnace at 90 °C for at least 3 h. The steps of the pellet preparation/pellet impregnation are summarized in Figure S1.

#### 2.1.3 Electrolytes

The water-containing organic electrolyte was prepared as reported by Söllinger et al.^20^ 2 g of Mg(ClO_4_)_2_·1.4H_2_O was dissolved in 10 mL of deionized water. Afterward, the solution was dried in a furnace at 90 °C overnight to obtain Mg(ClO_4_)_2_·3.1H_2_O. The obtained magnesium perchlorate was transferred into the glovebox, and 1.68 g were dissolved in 30 mL of ACN and used as the electrolyte. Electrochemical measurements performed with the cells built with this electrolyte are denoted as HVO-organic. For the water-based electrolyte, 1.93 g of Mg(NO_3_)_2_·H_2_O was dissolved in 15 mL H_2_O. Electrochemical measurements performed with the cells built with this electrolyte are denoted as HVO-aqueous.

#### 2.1.4 Electrode Preparation

The electrode preparation is similar to previous studies and described in more detail in the Supporting Information.^20,23^ The setup of the self-made battery cell is displayed in Figure S2.

### 2.2 Characterization

#### 2.2.1 Powder X-ray Diffraction (XRD)

The crystal structure of HVO was characterized by XRD on a Bruker D8 Advance diffractometer with a goniometer radius of 280 mm and equipped with a fast solid-state LynxEye detector. The sample was prepared on a zero-background single-crystal silicon sample holder. Afterward, the measurement was carried out using Cu–Kα_1,2_ radiation in the 2θ range from 5° to 90° with a step size of 0.015°. The structure of HVO was refined by TOPAS 6.0 (version 6.0, Bruker AXS Inc., WI, USA).^24^ The symmetry and lattice parameters given in the Inorganic Crystal Structure Database (ICSD) 80572 entry were used as the starting parameters.^25^

#### 2.2.2 Electron Microscopy

The morphology of HVO was analyzed with a Zeiss Ultra Plus Scanning Electron Microscope (SEM) using an in-lens secondary electron detector with an acceleration voltage from 3 kV to 10 kV. Furthermore, a JEOL-JEM-F200 TEM equipped with a cold field emission gun at an acceleration voltage of 200 kV, an high-angle annular dark-field (HAADF) detector used in scanning mode (STEM), and a large windowless JEOL Centurio EDX detector (100 mm^2^, 0.97 srad, energy resolution < 133 eV) was used. HVO for post-mortem TEM was gently washed with deionized water to remove potential residues of the separator and the electrolyte. The HVO samples were prepared by depositing an isopropanolic suspension of the compounds onto holey carbon copper grids. To remove carbon contaminants, TEM grids were cleaned gently in He plasma (Zepto, Electronic Diener) at 20 W for 45 s (base pressure: 0.08 mbar, pressure during plasma cleaning: 0.3 mbar).

##### 2.2.2.1 Galvanostatic cycling

Galvanostatic cycling was carried out using an 8-channel Astrol BAT-SMALL potentiostat. Specific capacities based on the mass load of the active material (HVO) were determined by galvanostatic discharge/charge cycling with a constant current (current density of 100 mA·g^–1^) in the voltage range of −1.1 to 0.2 V vs AC. To estimate the potential of HVO vs Mg/Mg^2+^, the Mg metal was used as the counter and reference electrode in the voltage range of 1.2–2.8 V vs Mg/Mg^2+^ at a current density of 50 mA·g^–1^.

##### 2.2.2.2 CV

CV was carried out in the voltage range of −1.1 to 0.2 V vs AC with a scan rate of 0.2 mV·s^–1^.

##### 2.2.2.3 GITT:

GITT was carried out during the discharge process of the IC until the potential reached −1.1 V vs AC at a current density of 100 mA·g^–1^ in an aqueous and organic electrolyte. The current flow time was set to 10 min in each case, while 15 min was selected for the relaxation times in between. The measurements of these cells were carried out on a Biologic SP-200 Single-Channel potentiostat.

#### 2.2.3 EIS

Before the EIS measurements were carried out, the cells were one-time discharged to −1.1 V and charged to 0.2 V vs AC at a current density of 100 mA·g^–1^ using the Astrol BAT-SMALL potentiostat. Afterward, an Autolab PGSTAT302N potentiostat featuring a response analyzer module (FRA2, Metrohm) was used for EIS measurements. The spectra were acquired with an amplitude of 10 mV in a frequency range of 0.1–200 kHz at potentials −0.5 V and −0.8 V vs AC for the organic and aqueous electrolytes. A time delay of 30 min was set before the start of a measurement to give the system time to adjust to the desired potential.

#### 2.2.4 Operando X-ray Diffraction

Similar to XRD measurements, operando XRD measurements were carried out using a Bruker D8 powder diffractometer. In addition to the parameters mentioned before, solar slits of 4° and an automatic *xyz* table were used. The 2θ range was set from 8° to 40° with a step size of 0.01° using an ECC-Opto-Std electrochemical cell setup by EL-CELL GmbH (Figure S3) for the measurements. A polymeric window was used as sealing and consisted of a polyethylene/ethylene-vinyl-alcohol/polyethylene/polyamide (PE/EVOH/PE/PA) foil (donated by Scheyer Verpackungstechnik GmbH). Galvanostatic measurements of the EL-CELLs were carried out on a BioLogic SP-50 Single-Channel potentiostat in the voltage range of −1.1 V to 0.2 V vs AC at a current density of 80 mA·g^–1^. The time of a single XRD scan was about 12 min.

## 3 Results and Discussion

**Figure 1:**
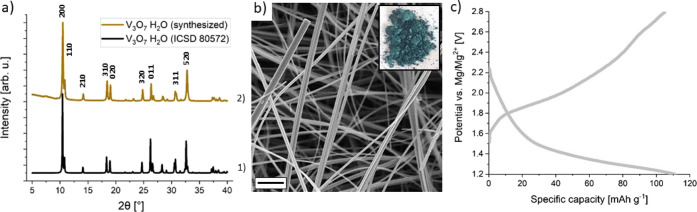
Structural, morphological, and electrochemical properties of V_3_O_7_·H_2_O (HVO). (a) Powder diffraction pattern of 1) HVO reference using the ICSD 80572 (black) and 2) synthesized HVO (brown). Indexed HVO patterns correspond to Bragg peak positions in the 2θ (°) range (space group *Pnam*). (b) SEM image of nanostructured HVO (scale bar: 1 μm). (c) IC of HVO vs Mg/Mg^2+^ in water-containing organic electrolyte.

In Figure 1, the structural, morphological, and electrochemical properties of V_3_O_7_·H_2_O (HVO) are presented. HVO belongs to the layered vanadium oxide class, where the intercalation occurs parallel to the *bc* plane.^26^

To confirm the phase purity of HVO, the diffraction pattern of the as-synthesized HVO is compared with the pattern of the orthorhombic HVO (ICSD 80572) (see Figure 1a). The matching patterns and the sharp diffraction peaks indicate phase purity and good crystallinity. The morphology was investigated by SEM, showing that HVO consists of fibers with diameters of 80 nm on average and lengths of a few micrometers (see Figure 1b). These characteristics are similar to HVO obtained in previous works.^27−30^ Furthermore, galvanostatic cycling of HVO was performed in water-containing organic electrolyte (0.25 M Mg(ClO_4_)_2_·3.1H_2_O) versus Mg metal at a current density of 100 mA·g^–1^ to identify the potential window of HVO vs Mg/Mg^2+^ (see Figure 1c). As shown, the potential window is 1.2–2.8 V vs Mg/Mg^2+^, demonstrating that HVO is a promising cathode material for magnesium-ion batteries. However, since the Mg metal is not (yet) compatible with aqueous electrolytes, we focus our research in this study on the intercalation mechanism in the cathode material using an AC pellet as the counter and reference electrode.

**Figure 2:**
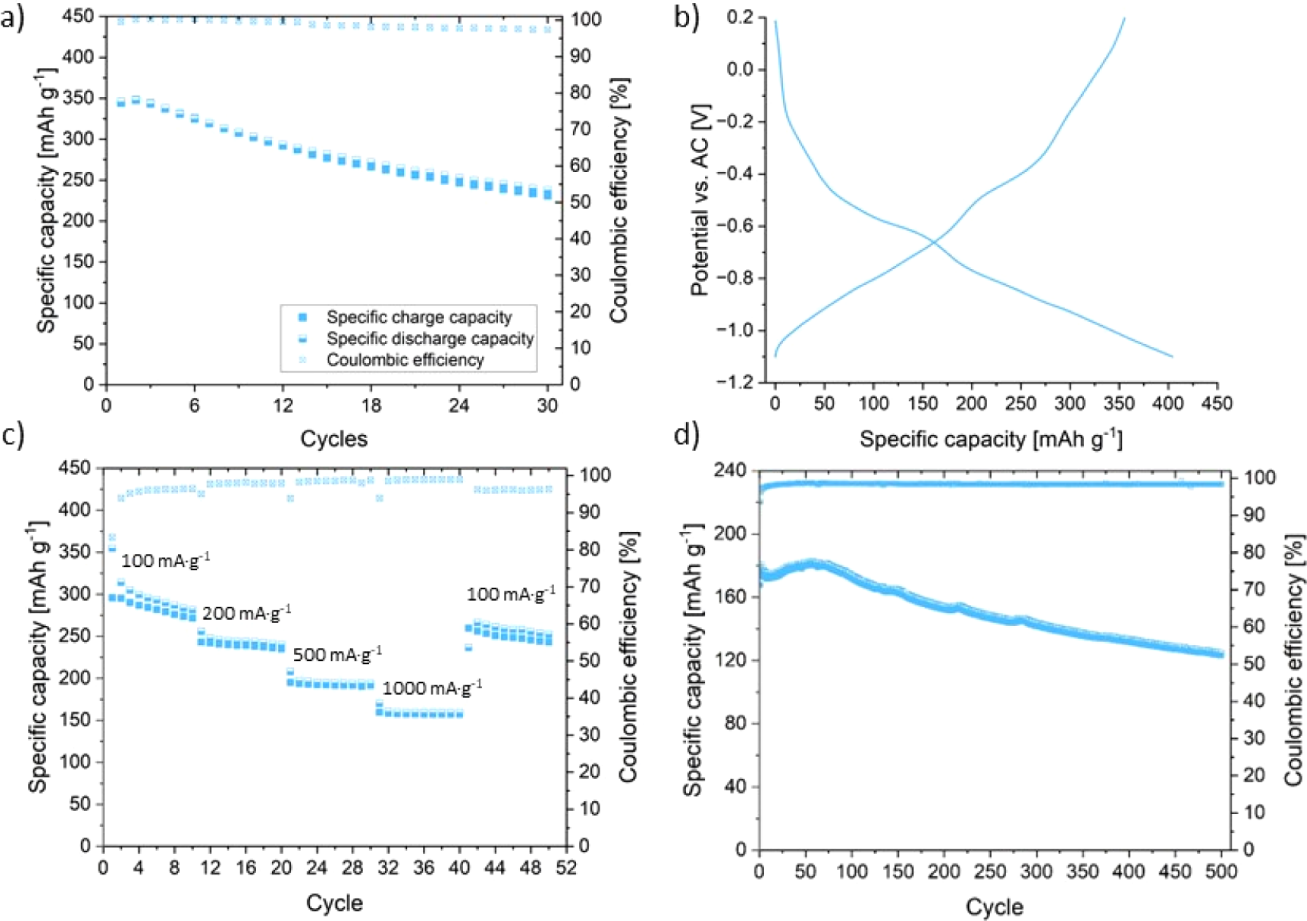
Galvanostatic cycling of HVO-aqueous in the potential range of −1.1 V to 0.2 V vs AC (at a current density of 100 mA·g^–1^). (a) Cycling performance and CE for the first 30 cycles. (b) Discharge/charge cycle of the IC. (c) C-rate measurements of HVO-aqueous at current densities of 100 mA·g^–1^, 250 mA·g^–1^, 500 mA·g^–1^, 1 A·g^–1^, and 100 mA·g^–1^ and (d) long-term galvanostatic cycling at a current density of 1 A·g^–1^.

Figure 2 shows the electrochemical performance of a HVO cathode vs an AC pellet as the counter and reference electrode in a 0.5 M Mg(NO_3_)_2_·H_2_O aqueous electrolyte (denoted as HVO-aqueous). HVO-aqueous-containing cells were first discharged to −1.1 V vs AC and afterward charged to 0.2 V vs AC at a current density of 100 mA·g^–1^. The galvanostatic cycling results displayed in Figure 2a show that HVO-aqueous reaches high specific capacities of ∼300 mAh·g^–1^ and stabilities, which are comparable to the performance of HVO in organic electrolytes in various battery types (see Table S2).^28,31−33^ The initial specific discharge capacity in Figure 2b reaches a remarkable 404 mAh·g^–1^ at a current density of 100 mA·g^–1^. This initial specific capacity is one of the highest obtained in aqueous Mg electrolytes at a practical current density compared to those of other cathode materials (see Table S1). However, after a supplementary cyclovoltammetric measurement (CV), the specific capacity dropped to 344 mAh·g^–1^ in the first cycle, followed by a slow decrease to 231 mAh·g^–1^ after 30 cycles (equal to 67% capacity retention). The Coulombic efficiency (CE) reached on average 98% over 30 cycles (see Figure 2a). C-rate measurements (Figure 2c) show that HVO-aqueous exhibits specific capacities of 296 mAh·g^–1^, 243 mAh·g^–1^, 195 mAh·g^–1^, 160 mAh·g^–1^, and 260 mAh·g^–1^ at current densities of 100 mA·g^–1^, 200 mA·g^–1^, 500 mA·g^–1^, 1 A·g^–1^, and 100 mA·g^–1^, respectively. The specific capacity of 260 mAh·g^–1^ after C-rate measurements demonstrates that HVO-aqueous exhibits adequate stability, since the retained capacity is comparable with HVO cathodes in organic electrolytes for various battery types (see Table S2) and shows higher specific capacities (see Table S1) than most reported cathode materials in aqueous Mg^2+^-containing electrolytes.^34,35^

Figure 2d demonstrates the performance of HVO-aqueous vs AC during long galvanostatic cycling at a high current density of 1 A·g^–1^. At this current density, an initial specific charge capacity of 168 mAh·g^–1^ was reached. After 500 cycles, the specific charge capacity retained a value of 123 mAh·g^–1^, corresponding to a capacity retention of 73% over 500 cycles, which is comparable with HVO cathodes in various battery types.^26,28,36^

**Figure 3:**
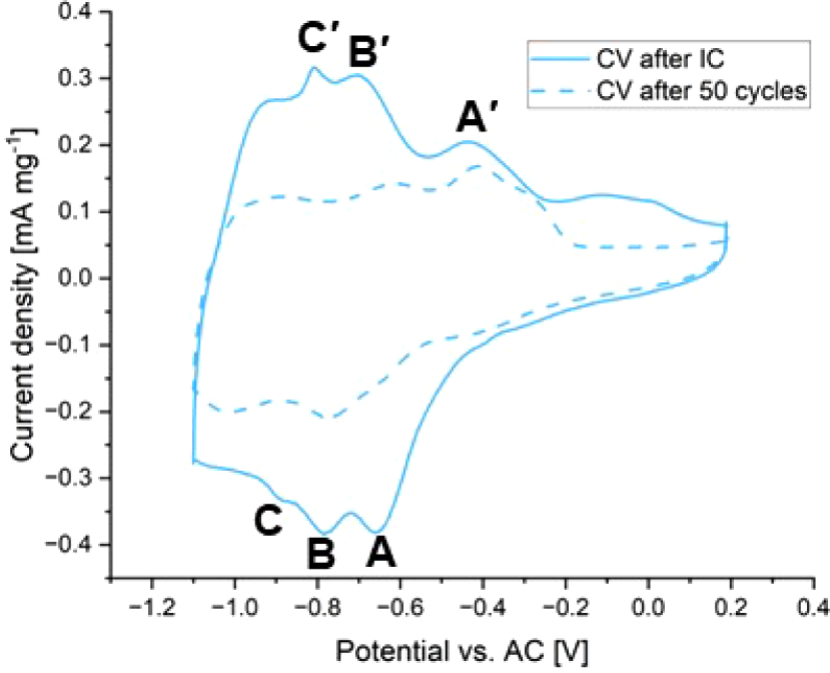
CV measurements of HVO-aqueous vs AC at a sweep rate of 0.2 mV·s^–1^ in the potential range of −1.1 to 0.2 V vs AC. The continuous line corresponds to the CV recorded after the IC, and the dashed line corresponds to the CV recorded after 50 galvanostatic cycles. The reduction peaks are labeled as A, B, and C, and the associated oxidation peaks are labeled as A’, B’, and C’.

To further investigate the electrochemical properties of HVO-aqueous vs AC, one CV cycle was acquired after the IC and one after 50 galvanostatic discharge/charge cycles, as displayed in Figure 3. The CV measured immediately after the IC shows three reduction peaks at −0.65 V (A), −0.8 (B), and −0.9 V (C) vs AC. In order to locate the corresponding oxidation peaks, CV cycles were acquired in the voltage ranges between 0.2 V and potentials after the respective reduction peaks A, B, and C, namely, −0.7 V (A), −0.85 V (B), −1.0 V (C), and −1.1 V (end of the discharge process) (see Figure S4). In the potential range −0.7 to 0.2 V vs AC, the redox pair A/A’ starts to form, suggesting that the peak at −0.4 V is the oxidation peak A’ associated with the reduction peak A. A “shoulder” of the redox pair B/B’ is formed after the cell was discharged to −0.85 V vs AC, followed by the formation of a more distinct redox pair of B/B’ during the discharge to −1.0 V vs AC. After fully discharging the cell to −1.1 V vs AC, a shift to lower potentials compared to the CV after the IC is visible for the redox pair B/B’. Therefore, the signature at −0.65 V was assigned to B’ and, in consequence, the peak at −0.8 V to C’. Interestingly, the redox pair C/C’ was not visible in the fourth CV cycle, suggesting that this redox couple is not very stable. Comparing in Figure 3 the CV after IC to the CV acquired after 50 galvanostatic cycles, a decrease of the peak area and of the peak heights of redox couples B/B’ and C/C’ are observed between −1.1 V and −0.6 V. Interestingly, the oxidation peak in the higher potential area (A’) is relatively stable with respect to position and area during the CV measurements at different potential windows and after 50 cycles (see Figure 3). This confirms the higher stability of the redox couple A/A’ in comparison to B/B’ and C/C’ in the lower potential range.

In order to investigate these stability aspects further, the CVs of HVO-aqueous are compared to Mg^2+^ insertion into HVO vs AC in a water-containing organic electrolyte (see Figure S5).^20^ HVO-aqueous contains a larger number of distinct signatures. Specifically, the redox peaks B/B’ and C/C’ appear as additional features at lower potentials. In the higher potential range (signatures A/A’), the CVs of HVO-aqueous and HVO in a water-containing organic electrolyte exhibit similar characteristics. When interpreting these differences, it is important to take into account that HVO-aqueous and HVO in the water-containing organic electrolyte do not differ in the electrolyte alone but potentially in the ion content as well because of the presence of Na^+^ in the binder in the case of HVO-aqueous. Indeed, it has already been shown that Na-CMC as a binder introduces Na^+^ into electrochemical cells.^15,37^ Recently, Zhang et al. reported that sodium deficiencies in NaTi_2_(PO_4_)_3_ used as an anode material in a SIB were compensated by the presence of Na-CMC.^15^ Therefore, it is relevant to compare our results to the CVs of HVO vs Na/Na^+^. Previous publications report that CVs of HVO vs Na/Na^+^ in an organic electrolyte exhibit two reduction and two oxidation peaks.^17,33^ Therefore, we suggest that the additionally observed redox peaks B, B’ and C, C’ located at lower potentials in HVO-aqueous are connected to the (co)intercalation of Na^+^, with Na^+^ entering the system via Na-CMC in the AC pellet, while the stable redox couple at higher potentials A/A’ with characteristics similar to Mg^2+^ insertion in water-containing organic electrolyte is associated mainly with the intercalation of Mg^2+^ in HVO-aqueous. Based on this assumption, we deduce that the area that experiences higher loss after 50 galvanostatic cycles (B/B’ and C/C’) is associated with the intercalation of both ion species compared to the area attributed to Mg^2+^ intercalation (A/A’). A similar observation was made by Wang et al., where the deintercalation of Li^+^ in Li_3_V_2_(PO_4_)_3_@C in a Mg ion cell led to additional plateaus/peaks during initial galvanostatic charge cycles.^10^ However, similar to this work, the influence of Li^+^ (the minority ion species) was no longer detectable after several charge and discharge cycles, probably due to the higher amount of Mg^2+^ in the electrolyte compared to the amount of Li^+^ in the cathode material, as suggested by the authors. Therefore, the decrease of the peak area, which we attributed to the cointercalation of Na^+^ and Mg^2+^, is probably related to the lower concentration of Na^+^ in the electrolyte compared to Mg^2+^.

**Figure 4:**
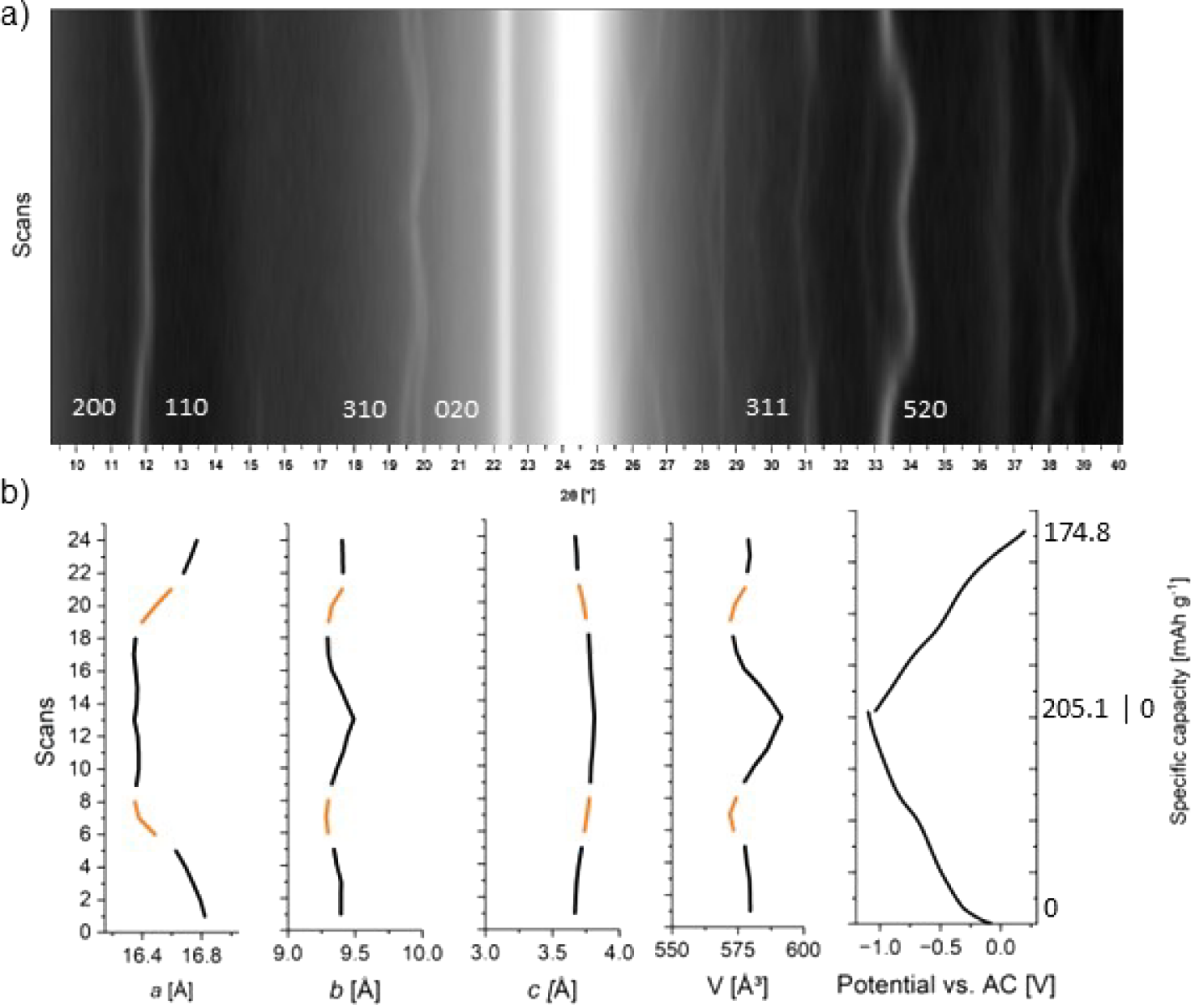
Operando XRD measurement during the first galvanostatic cycle of HVO-aqueous at a current density of 80 mA·g^–1^ between −1.1 and 0.2 V vs AC. (a) 2D diagram of the operando XRD pattern (*y*-scale is set to logarithmic intensities). (b) Refined lattice parameters of intercalated Mg^2+^ and Na^+^ in HVO-aqueous. Orange lines indicate values for the majority phase in the two-phase region, while black refers to solid solution processes.

To further confirm the hypothesis of the predominant intercalation of Mg^2+^ at the beginning of the discharge process followed by the intercalation of Mg^2+^ and Na^+^ in the lower potential range, operando XRD measurements were carried out and are displayed in Figure 4. Since the initial Coulombic efficiency is often lower in systems with AC as the counter electrode compared to the subsequent cycles because of the activation of the AC pellet, the operando XRD measurement of the first cycle after the IC is discussed in the main part, whereas the IC is analyzed in the Supporting Information (see Figure S6). Figure 4a shows the 2D plot of the XRD patterns recorded during a full discharge and charge process. The 2θ range between 21.5° and 25.5° corresponds to the background signal of the PE/EVOH/PE/PA foil (polymeric window) used for sealing. The lattice parameters of HVO are obtained by XRD pattern fitting of each scan via TOPAS and are shown in Figure 4b together with the respective specific capacities measured at a current density of 80 mA·g^–1^ between −1.1 and 0.2 V vs AC. For HVO, the intercalation of 1 mol Mg^2+^ into HVO corresponds to a theoretical specific capacity of ∼190 mAh·g^–1^ and ∼95 mAh·g^–1^ for 1 mol Na^+^. The lower specific capacity and CE compared to the electrochemical properties shown in Figure 2 are presumably caused by differences in the cell setup, i.e., the lower tightness of the EL-CELL because of the foil sealing or the use of a steel grid instead of a steel plate as the current collector. Although the specific capacity is decreased, we assume that the measurements are representative of the intercalation process of Mg^2+^ and Na^+^ into HVO in an aqueous electrolyte.

Analyzing the intercalation mechanisms, we find that the discharge process starts with a solid solution process, followed by a two-phase process (indicated by orange lines in Figure 4b), and continues afterward with a solid solution process until the end of the discharge process. The transition from a solid solution process to a two-phase process and back to a solid solution process is shown for Bragg peak 520 in Figure S7 in more detail. A similar behavior has been reported by operando XRD measurements of HVO with respect to Mg^2+^ intercalation in a water-containing organic electrolyte.^20^ Based on these findings, a (de)intercalation reaction equation of Mg^2+^, Na^+^, and HVO is proposed (eq 1), leading to a decrease of V^5+^ and an increase of V^4+^ during the intercalation (see Figure S8), which is also observed for our measurements and comparable to Li^+^ intercalation in HVO.^38,39^
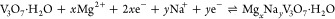
1

In the beginning of the discharge process, the lattice parameters *a* and *b* decrease compared to the values before the start of the discharge process (see Table 1). Continuing with the discharge process, *a* remains relatively constant, while *b* and *c* increase, reaching their maximum values at the end of the discharge process. This behavior leads to an increase in the unit cell volume.

**Table 1:**
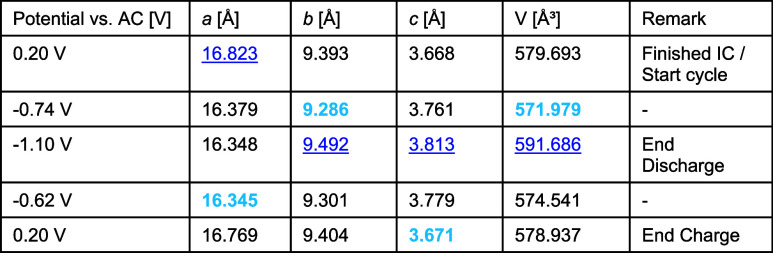
Minimum (Highlighted in Bold Light Blue) and Maximum (Highlighted in Underlined Dark Blue) Lattice Parameters and Unit Cell Volume for Mg^2+^ and Na^+^ Intercalation during the First Discharge/Charge Process of HVO-Aqueous vs AC

During the charge process, the values *b*, *c*, and V decrease again while *a* remains fairly stable until it expands toward the end of the charge process. Comparing the lattice parameters obtained after the IC and after the first cycle, we find that the values differ only slightly (below 0.35%), indicating high structural reversibility. The high reversibility is confirmed via Raman and ex-situ XRD measurements (see Figures S9 and S10). In addition, we find that no byproducts or irreversible reactions are detectible after a full cycle in comparison to the uncycled cell. The absence of V in the EDX spectra of the separator after the initial discharge and charge cycle suggests that vanadium is not dissolved during the cycling process (see Figure S11).

With respect to unit cell volume changes, Söllinger et al. reported recently that the intercalation of Na^+^ into HVO in an organic electrolyte led overall to an expansion of the unit cell volume, mostly due to the comparatively large ionic radius of Na^+^, and exhibited exclusively solid solution processes.^21^ In contrast to Na^+^, the intercalation of other (smaller) ions such as Li^+^, Mg^2+^, or Zn^2+^ led to a contraction of the HVO structure and took place via both solid solution and two-phase processes. Table S3 shows in detail the maximum and minimum values of the HVO lattice parameters during the intercalation of Mg^2+^ and/or Na^+^ in different electrolytes. Therefore, the unit cell expansion observed in HVO-aqueous confirms the assumption that (additional) intercalation of Na^+^ into HVO-aqueous solutions took place. In addition, the observation of two-phase processes in HVO-aqueous solutions points to the intercalation of Mg^2+^.

To summarize, the observed behavior of HVO-aqueous during intercalation comprised the signatures of both ion types, Mg^2+^ and Na^+^, suggesting a hybrid cell. At the beginning of the discharge process, i.e., at higher potentials, the intercalation of Mg^2+^ into HVO seemed to be dominating, since abrupt changes of various Bragg peaks and the formation of the two-phase process are visible, signatures that are connected to Mg^2+^ intercalation as suggested in previous publications.^20^ At lower potentials, the intercalation of Na^+^ is more important, leading to an expansion of the unit cell volume unique for Na^+^ intercalation into HVO compared to the intercalation of other ions such as Li^+^, Zn^2+^, or Mg^2+^, as shown in our previous work.^21^ These results are in agreement with our hypothesis based on cyclovoltammetry.

**Figure 5:**
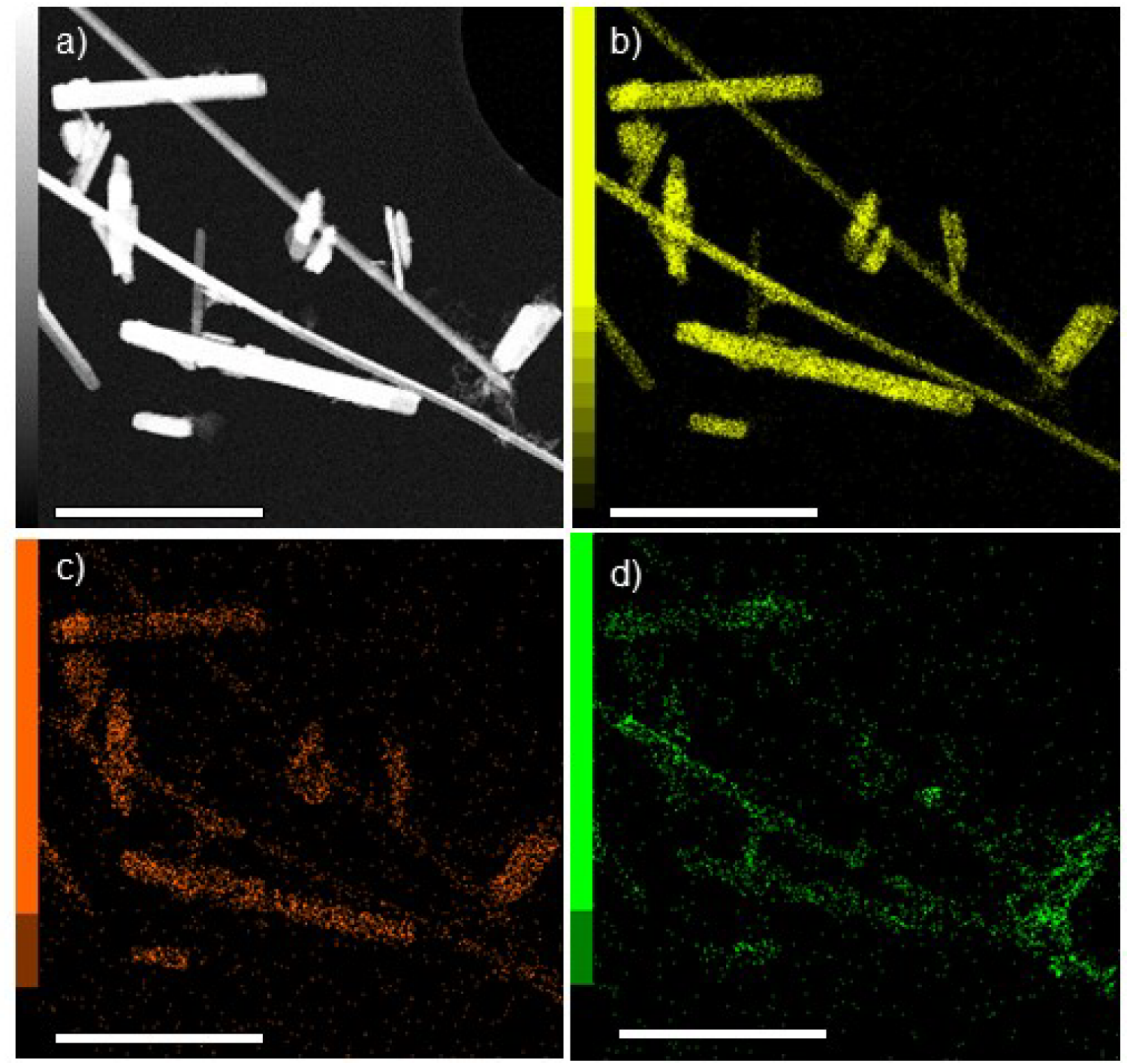
STEM image and EDX intensity maps of post-mortem HVO-aqueous (scale bar = 1 μm). (a) HAADF image. EDX intensity maps of (b) V–K edge, (c) Na–K edge, and (d) Mg–K edge.

To verify the presence of Mg^2+^ and Na^+^ in HVO after galvanostatic discharge and the reversibility of the intercalation process after a full cycle, post-mortem STEM/EDX measurements were performed on HVO and are displayed in Figures 5 and S12. The HVO samples for post-mortem TEM studies were obtained from a cell without any additives or binders at the cathode. In consequence, the specific capacity in these cells was limited to ∼110 mAh·g^–1^. Figure 5a shows an HAADF image of HVO nanofibers after being discharged in an aqueous electrolyte. Comparing Figure 5a to the V–K EDX intensity map in Figure 5b, an even distribution of V is visible and confirms that the nanofibers are made up of HVO. In Figure 5c,d, the Na–K and the Mg–K edge EDX intensity maps are shown, respectively, suggesting a homogeneous distribution of Na^+^ and Mg^2+^ in the HVO nanofibers. Furthermore, Figure S13 includes a table listing the atomic ratios between V, Mg, and Na. Compared to Mg^2+^ insertion into HVO in water-containing organic electrolyte, which showed that intercalated Mg^2+^ mainly accumulated at the surface of the HVO nanofibers, the distribution of Mg^2+^ is more homogeneous in the fibers of HVO-aqueous, i.e., Mg^2+^ diffuses into the interior of the fibers.^20^ This raises the question of whether it is rather the electrolyte composition or the hybrid intercalation of two ion species (Mg^2+^ and Na^+^) that is responsible for this observation.

**Figure 6:**
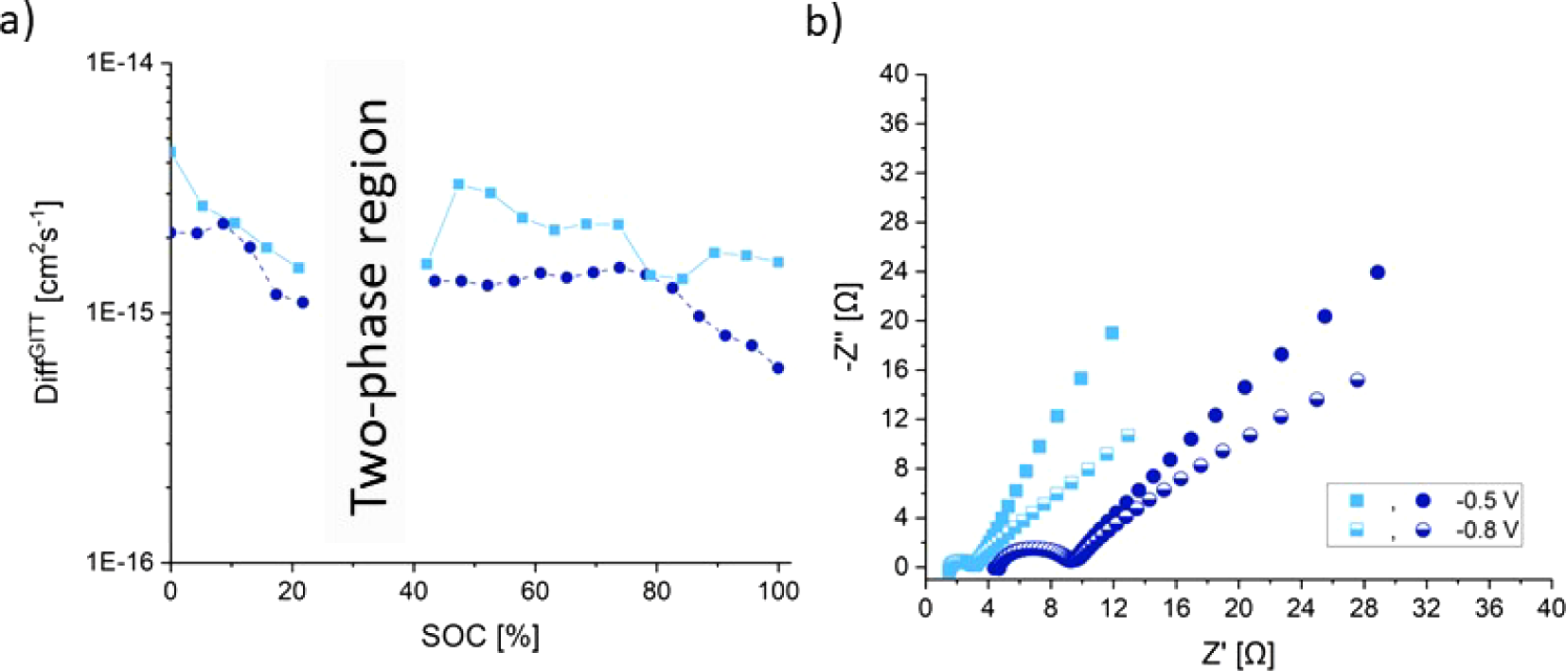
GITT and EIS measurements of HVO-aqueous (light-blue) and HVO-organic (dark-blue) vs AC at a current density of 100 mA·g^–1^. (a) Chemical diffusion coefficients as a function of the state of charge (SOC) during the initial discharge cycle to −1.1 V vs AC. (b) Nyquist plots obtained from EIS measurements at potentials −0.5 V and −0.8 V vs AC.

To differentiate between electrolyte and hybrid intercalation effects, further electrochemical characterization was carried out by GITT and EIS (see Figure 6) in cells with the same cell setup (including a Na-CMC binder in all cells) but different electrolytes. The cell using an AC pellet with Na-CMC as the potential Na^+^ source and a water-containing organic Mg^2+^ electrolyte is denoted HVO-organic, while the same cell with a purely aqueous electrolyte is further referred to as HVO-aqueous. The equation for the determination of the diffusion coefficient *D* as displayed in Figure 6a is described in the Supporting Information.^40^ The corresponding potential feedback curves are shown in Figure S14. Interpreting these results, we take into account that the equation used to calculate the diffusion coefficient is simplified and is based on several approximations. Furthermore, the calculation of the diffusion coefficient was limited to SOCs in the single-phase regions, i.e., regions where solid solution processes take place.

During the initial discharge, the obtained diffusion coefficients were on average 2.5 × 10^–15^ and 1.7 × 10^–15^ cm^2^·s^–1^ before the start of the two-phase region and 2.1 × 10^–15^ and 1.1 × 10^–15^ after the two-phase region for HVO-aqueous and HVO-organic, respectively (see Figure 4). The most pronounced difference was observed at the end of the discharge at SOCs larger than 80%, where HVO-organic exhibited lower diffusion coefficients compared to HVO-aqueous. However, HVO-organic exhibited slightly more potential feedback curves, which is an indication of higher specific capacities (Figure S14).

In order to further study ion insertion into HVO, EIS was carried out after the IC at −0.5 V and −0.8 V vs AC. The potentials were chosen to compare resistance differences in a nearly empty cathode with a cell in a SOC of ∼70%. The corresponding Nyquist plots in Figure 6b show one depressed semicircle followed by a spike with a takeoff angle lower than 45° for HVO-aqueous and HVO-organic at high frequencies. These features have been observed before in previous EIS measurements of HVO in various battery types, mainly in organic electrolytes.^17,41^ The ohmic resistance (Rs) is estimated by taking the distance of the coordinate origin (0 Ω) to the beginning of the first semicircle, which is an indication of the electrical resistances of the cell components and the conductivity of the electrolyte. For HVO-aqueous and HVO-organic, Rs shows values between 1 and 5 Ω. We assume that the following semicircle corresponds to the charge transfer in the active material and is simulated with an RC circuit (*R*_ct_). The resistances obtained from the diameter of the semicircle show values of ∼3 Ω for HVO-aqueous and ∼7 Ω for HVO-organic for the potentials −0.5 V and −0.8 V vs AC. Compared to the literature, the resistances observed in this work are much lower. *R*_ct_ values for HVO in other battery types such as LIBs in dry organic electrolyte show typical resistances of ∼200–400 Ω.^17,36,41^ The angles after the semicircle, simulated with the Warburg impedance (*W*), are smaller at lower potentials but in the same range for HVO-aqueous and HVO-organic. Although the resistances are low in both cases compared to literature values obtained mainly for cells containing dry organic electrolytes, HVO-aqueous shows slightly lower values compared to HVO-organic. This effect is more pronounced if the cells are more often discharged and charged before the start of the EIS measurements (see Figure S15). The reason for these observations lies probably in the shielding effect of water, which facilitates the intercalation of doubly charged ions like Mg^2+^. It is conceivable that the lower water content in the organic electrolyte compared to the aqueous electrolyte leads to a reduced shielding effect during ion insertion.

## 4 Conclusions

We studied the performance and the (co)intercalation mechanism of Mg^2+^ and Na^+^ into V_3_O_7_·H_2_O (HVO) as a cathode material in an aqueous electrolyte. The specific discharge capacity reached 404 mAh·g^–1^ at a current density of 100 mA·g^–1^, which is one of the highest reported for Mg^2+^/Na^+^ intercalation in an aqueous electrolyte. At a high current density of 1 A·g^–1^, a specific discharge capacity of 168 mAh·g^–1^ was achieved. After 500 galvanostatic discharge and charge cycles, a specific capacity of 123 mAh·g^–1^ (73%) was retained, which is comparable with the performance of HVO as a cathode material in less sustainable and less safe battery types, e.g., HVO vs Li/Li^+^ in organic electrolytes. The hybrid character of the cell was confirmed by CV, operando XRD, and STEM/EDX measurements. Cyclovoltammetry measurements show the presence of three redox pairs. Compared to the intercalation of Mg^2+^ in HVO in a nonhybrid cell, two additional redox pairs are present.

To investigate the insertion mechanism in more detail, operando XRD measurements were carried out, indicating that the intercalation of Mg^2+^ in HVO is dominant at higher potentials (at the beginning of the galvanostatic discharge process), followed by the intercalation of Na^+^ starting at ∼−0.8 V vs AC. The presence of Mg^2+^ and Na^+^ in HVO after the galvanostatic discharge process was further confirmed by STEM/EDX measurements, proving that both ion types are distributed in the HVO nanofibers.

To explore the influence of the aqueous nature of the electrolyte on the electrochemical properties of HVO, GITT and EIS measurements were carried out in an aqueous electrolyte and in water-containing organic electrolyte. HVO shows similar diffusion coefficients in both electrolytes (1.1 × 10^–15^ to 2.5 × 10^–15^ cm^2^·s^–1^). EIS measurements indicate extremely low charge transfer resistances in both electrolytes (3–7 Ω), about 2 orders of magnitude lower than in organic electrolytes, e.g., in LIBs or SIBs. In addition, HVO in an aqueous electrolyte shows resistances slightly lower than those in a water-containing organic electrolyte. These findings are explained by the fact that water shields the ion charge during intercalation.

In summary, our results demonstrate that HVO is an excellent cathode material for Mg^2+^/Na^+^ cointercalation in a sustainable aqueous hybrid cell with state-of-the-art performance, even when compared to organic electrolyte systems. In order to enable the use of sustainable aqueous hybrid cells for practical applications, such as stationary electrical energy storage, further research is needed to find suitable cathodes and anodes for Mg^2+^ and/or Na^+^ cells, potentially inspired by promising electrode systems operational in organic electrolytes.^42−44^

## Supporting Information Available

Detailed descriptions of the used chemicals, the synthesis of HVO and the electrode preparation, the setup of the battery cells, further electrochemical measurements (CVs, EIS), operando XRD, ex-situ XRD and Raman measurements, and EELS measurements and STEM/EDX images (PDF)
