# Supplementary material for: V3O7·H2O as a Cathode Material for Aqueous Mg2+/Na+ Hybrid Electrochemical Cells
Source: ACS Omega. 2025 Mar 4;10(10):10152–61. doi: 10.1021/acsomega.4c08983 (PMC11923679; doi:10.1021/acsomega.4c08983)
Supplement: Supplementary file 1 — ao4c08983_si_001.pdf [file ao4c08983_si_001.pdf]

Supplementary Information (SI):

$V_3O_7 \cdot H_2O$ as cathode material for aqueous Mg^{2+}/Na^+ hybrid electrochemical cells

Daniela Söllinger,[a*] Julie Lam Chen,[a] Jakub Zalesak,[a] Jakob Praxmair,[a] Simone Pokrant[a]

Authors:

Dr. D. Söllinger, J. Lam Chen, Dr. J. Zalesak, J. Praxmair, Prof. S. Pokrant*

[a]*Chemistry and Physics of Materials, University of Salzburg, 5020 Salzburg, Austria*

*Corresponding Author:

E-Mail: Daniela.soellinger@plus.ac.at Tel.: +43 662 8044 6225

Experimental section:

Chemicals: Carbon black (99+ %), L(+)-Ascorbic acid (99+ %), magnesium perchlorate ($Mg(ClO_4)_2 \cdot H_2O$, ACS), oxalic acid ($H_2C_2O_4$, 98 %), polyvinylidene fluoride (PVDF) and vanadium pentoxide (V_2O_5, 99.2 %), were purchased from Alfa Aesar. Anhydrous acetonitrile (ACN, 99.8 %), magnesium nitrate hexahydrate ($Mg(NO_3)_2 \cdot 6H_2O$, for analysis), Na-CMC ($C_{28}H_{30}Na_8O_{27}$, low viscosity), Tetrahydrofuran (THF, for analysis) and Whatman glass fiber filters (grade GF/D) were purchased from Merck. Activated carbon (AC, activated charcoal) and toluol were purchased from Avantor. SBR-Binder (BM-400B) was donated by ZEON Corporation, Specialty Materials Div. Graphite oxide (~60 % H_2O) was purchased from Belenos. Magnesium foil (purity 99.8 %) was purchased from Hauner GmbH & Co. KG. All reagents were used as received.

Synthesis of nanostructured $V_3O_7 \cdot H_2O$ (HVO): The nanostructured precursor V_2O_5 was synthesized following the synthesis reported by Zhan *et al.* without the addition of $Cr(NO_3)_3$.[1] First, 10.4 g of $H_2C_2O_4$ were dissolved in 100 ml of deionized water at room temperature in a three-neck flask. Next, 5 g of V_2O_5 were added, followed by stirring for 3 h and further stirring at 80 °C for 5 h. The obtained solution was dried at 100 °C for 10 h and afterwards calcinated at 400 °C for 10 h.

Nanostructured HVO was synthesized as reported by Söllinger *et al.* [2,3] 90 mg ascorbic acid were dissolved in 25 ml deionized water. 2 g of the obtained V_2O_5 powder were added to this solution followed by stirring under reflux at 110 °C for 16-17 h in a round bottom flask. Afterwards, the suspension was transferred into an autoclave with a 100 ml polytetrafluoroethylene (PTFE) inlet. Further 25 ml of deionized water were added to the suspension to half-fill the inlet before the start of the hydrothermal process. The autoclave was afterwards put in a furnace at 220 °C for 6 h. The obtained precipitate was washed three times with deionized water and once with isopropanol via a centrifuge and dried at 80 °C for 3 h.

Electrode preparation: The electrodes were prepared in a mixture of 60:30:10 (wt%) between active mass (HVO), carbon black and PVDF. [2,3] First, 60 mg of HVO and 30 mg of carbon black were ground for at least 10 min. In between, 10 mg of PVDF was dissolved in 3 ml THF/Toluol (2:1, volume ratio). Before the dissolved solution was added to the ground compounds, the weight of the stainless-steel current collector (area 1.3 cm^2) was measured. Afterwards, the solution and the compounds were further ground until a honey-like viscous texture was obtained. Immediately afterwards, the slurry was drop casted on a current collector. Finally, the electrodes were dried in a vacuum furnace at 90 °C for at least 3 h. Cells with water-containing, organic electrolyte were assembled in an argon-filled glove box (< 0.1 ppm H_2O and < 0.1 ppm O_2). As separator, Whatman glass fiber filters with a diameter of 16 mm were used. AC-pellets were used as counter and reference electrode. In this configuration, the mass ratio between the active mass and the AC-pellets was set 1:50. The aqueous electrolyte was impregnated into the AC-pellet using a vacuum pump to remove the air inside the pellets. Afterwards, the pellet was again used as counter and reference electrode in the cell.

For *operando* XRD measurements, a stainless-steel grid was poured with the honey-like viscous HVO cathode texture. The active mass was adjusted to a minimum of 3 mg to achieve appropriate intensities during the measurements. The mass ratio between the active material and the AC-pellet was 1:20. Please note that we used a self-customized lid.

Nitrogen-sorption: N_2-sorption measurements were carried out using a Micromeritics ASAP 2420 sorption apparatus at -196 °C. HVO was degassed at 80 °C for 12 h and afterwards the specific surface area (SSA, m^2 g^{-1}) was determined by the BET method.

Table S1. Comparison of the electrochemical performance of representative cathode materials in aqueous Mg^{2+} or Na^{+} containing electrolytes.

**Cathode material**	**Reference (RE) / Counter electrode (CE)**	**Electrolyte**	**Initial specific capacity [mAh·g^{-1}]**	**Current density [mA· g^{-1}]**	**Potential [V]**	**Literature**
δ-MnO_2 @MWCNTs/ CC	Activated carbon	0.5 M $MgSO_4$	247	50 mA· g^{-1}	-1.0 to 0.2 V	*Nanotech no-logy*, **2021**, *32* 445401
MgVO/C	RE: Saturated calomel electrode (SCE) CE: Pt foil	3 M $Mg(NO_3)_2$	65	200 mA· g^{-1}	-1.0 to 1.0 V	*Nanomate rials* **2022**, *12(16)*, 2767
Milled Mn_3O_4	RE: Ag/AgCl CE: Activated carbon	2 M $MgSO_4$	103	100 mA· g^{-1}	-1.0 to -0.2 V	*ChemElec troChem*, **2018**, *5*, 2789-2794
$Li_3V_2(PO_4)_3$/C	RE: Ag/AgCl CE: Activated carbon	4 m Mg $(TFSI)_2$	115	100 mA· g^{-1}	-0.2 to 1.0 V	*ACS Cent. Sci.* **2017**, *3, 10*, 1121–1128
$Na_4Mn_9O_{18}$	RE : Hg/Hg_2SO_4 CE : Activated carbon	1 M Na_2SO_4	45	~10 mA g^{-1}	-0.4 and 0.25 V vs. Hg/Hg_2SO_4	*Electroch em. commun*. **2010**, 12 463–466
Aerosol-assisted chemical vapor deposition V_2O_5	RE: Ag/AgCl/KCl CE: Pt foil	0.075 M $MgCl_2$	300	15 A· g^{-1}	-1.5 to 1.0 V	*J. Electroch em. Sci. Eng.*, **2020**, *10(3)*, 257-262
V_2O_5 nanowires	RE: AgCl/Ag CE: Activated carbon	0.8 M $Mg(TFSI)_2$-100%H_2O	245	50 mA· g^{-1}	−0.75 to 0.75 V	*Adv. Funct. Mater.* **2022**, *32*, 2110674
V_3O_7· H_2O	Activated carbon	0.5 M $Mg(NO_3)_2$	404	100 mA·g^{-1}	-1.1V – 0.2 V vs. Active Carbon	This work

Table S2. Comparison of the electrochemical performance of HVO/HVO modifications in organic or aqueous electrolytes from previous studies for LIBs, SIBs, MIBs, ZIBs and KIBs.

**HVO/HVO modification**	**Initial specific capacity [mAh· g^{-1}]**	**Current density [mA· g^{-1}]**	**Potential [V]**	**Battery type**	**Literature**
Li-HVO-rGO via hydrothermal process	371	100	1.70 – 3.90 V vs. Li/Li^+	LIB	*ChemElectroChem* **2021**, *8*, 4223–4232
$V_3O_7{\cdot}H_2O$@C Nanoribbons via high-temperature mixing method	262	500	3.75 – 1.50 V vs. Li/Li^+	LIB	*ACS Appl. Mater. Interfaces* **2017**, *9*, 17002−17012
Single crystal HVO nanobelts via hydrothermal process	409	20	1.50 – 3.75 V vs. Li/Li^+	LIB	*Electrochim. Acta.* **2009**, *54*, 1115-1118.
Composite material HVO/PEDOT:PSS	300	300	1.50 – 3.50 V vs. Li/Li^+	LIB	J. Mater Sci., **2023**, *58*, 4565–4575
rGO HVO film via hydrothermal process	377	100	1.50 – 3.50 V vs. Li/Li^+	LIB	*Solid State Ionics*, **2019**, *329*, 74–81
HVO NW/Graphene composite via hydrothermal process	394 (in an aqueous electrolyte)	100	0.20 – 1.60 V vs. Zn/Zn^{2+}	ZIB	*Adv. Energy Mater*. **2018**, *8*, 1800144
HVO/Mxene composite	365	200	0.20 – 1.60 V vs. Zn/Zn^2	ZIB	*Chemical Engineering Journal*, **2021**, *405*, 126737
HVO	168	5	-1.6 – 0.8 V vs. AC	KIB	*ChemistrySelect* **2019**, *4*, 11711–11717
Flexible additive free HVO nanowire membrane via hydrothermal process	160 (after 100 cycles)	10	1.50 – 4.00 V vs. Na/Na^+	SIB	*New J. Chem.*, **2014**, *38*, 2075-2080
HVO nanowires via hydrothermal process	231	10	-1.4 – 1.0 V (vs. AC)	MIB	*Chem. Mater.* **2018**, *30*, 7464−7472

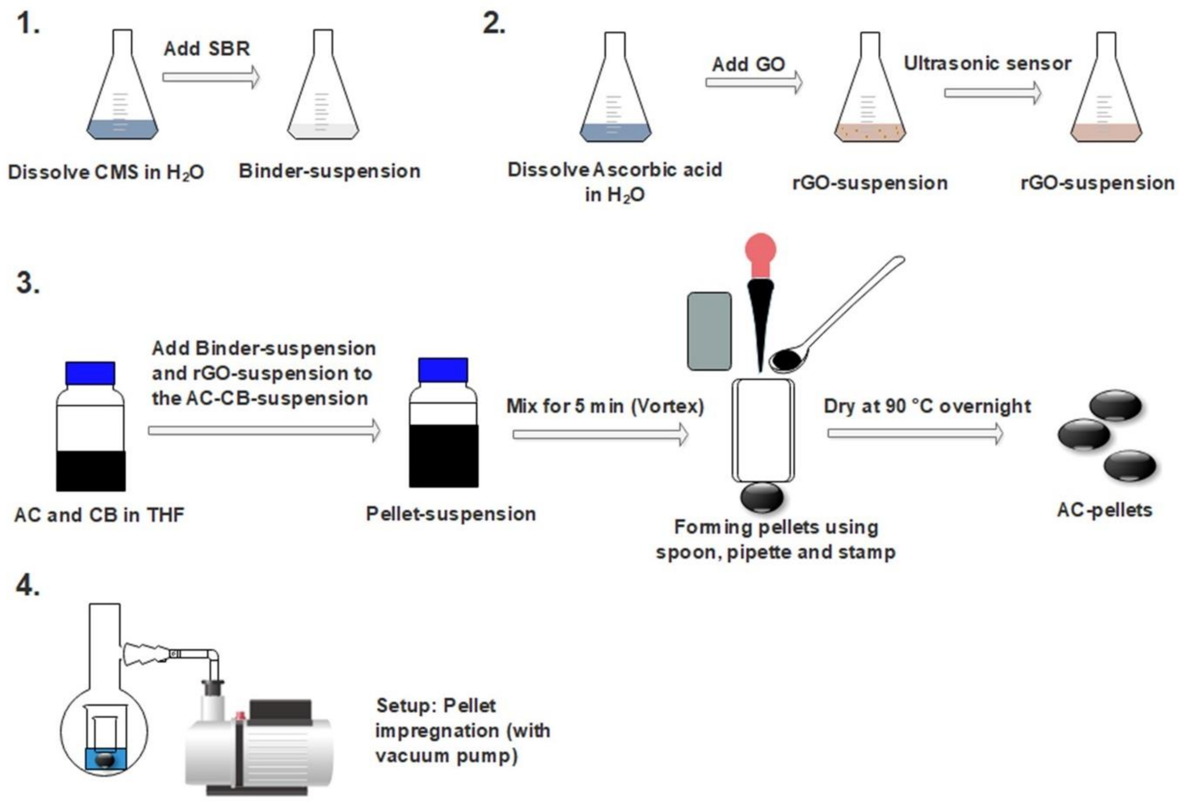

Figure S1. Preparation steps for AC pellets; 1) Dissolve CMS-Binder in H_2O followed by the addition of the SBR-Binder 2) Dissolve ascorbic acid in H_2O, followed by the addition of GO and the use of the ultrasonic sensor. 3) AC and CB are mixed in THF followed by mixing it for 5 minutes. Afterwards suspensions 1) and 2) are added to 3) and mixed again. Finally the pellets are pressed using a shell, a current collector with the desired diameter and a press tool. The as-prepared pellets are dried overnight at 90 °C in a furnace. After that the impregnation with the desired electrolyte can be done through a vacuum pump.

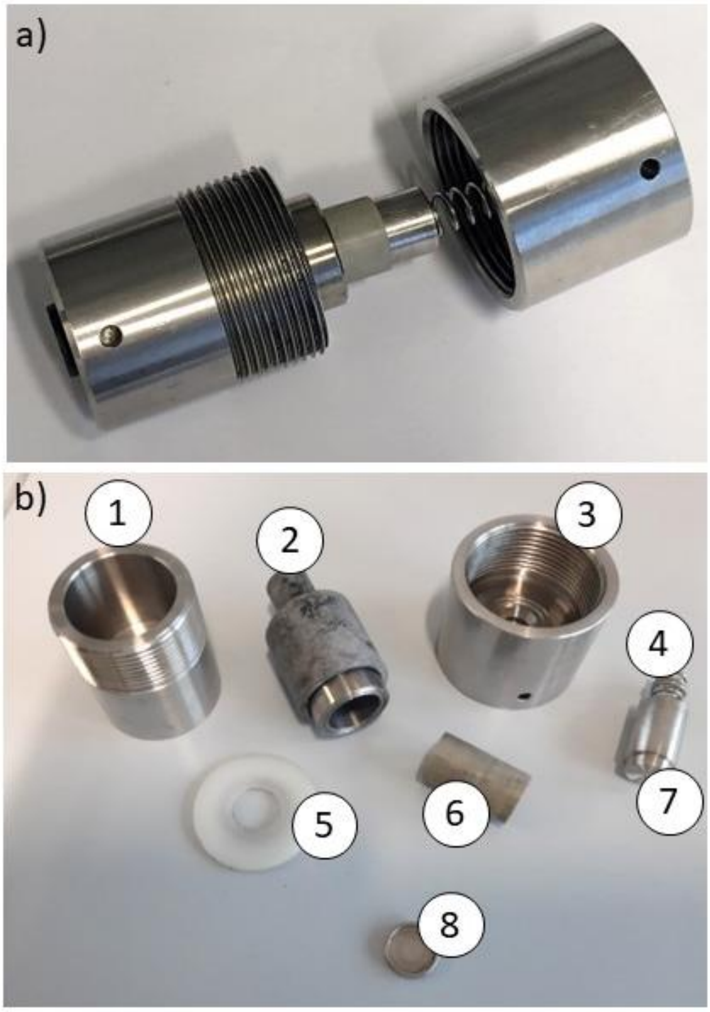

Figure S2. Photographs of battery cell: a) assembled battery cell, b) components of the battery cell (1) housing (top), (2) stamp, (3) housing (bottom), (4) spring, (5) sealing ring, (6) shielding, (7) stamp and (8) steel current collector.

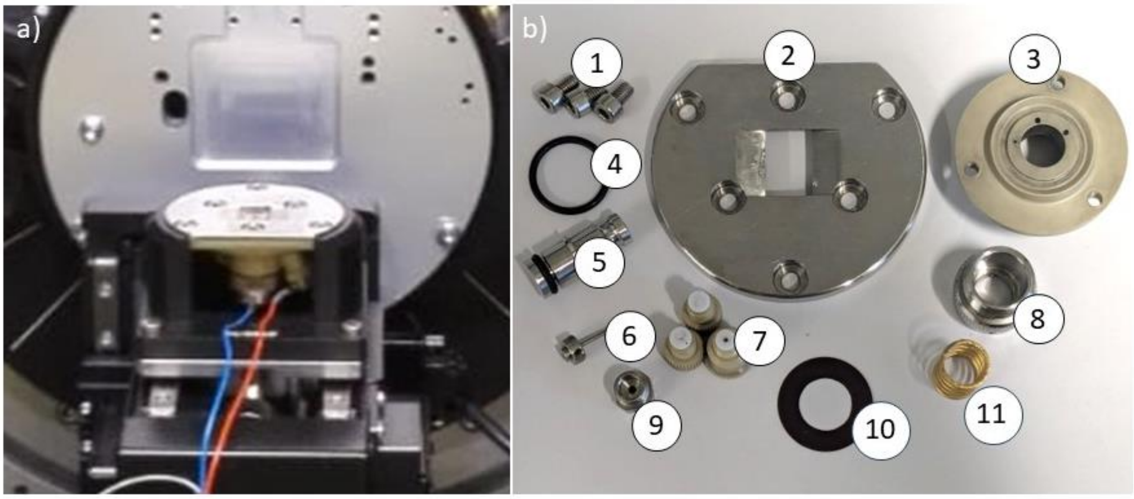

Figure S3. Set-up of the EL-CELL; a) assembled EL-CELL, b) single components of the EL-CELL: (1) screw, (2) lid, (3) base, (4) sealing ring, (5) piston with sealing ring, (6),(7) electrode pin/plug, (8) setscrew, (9) screw cap for electrode pin/plug, (10) pressure ring and (11) spring

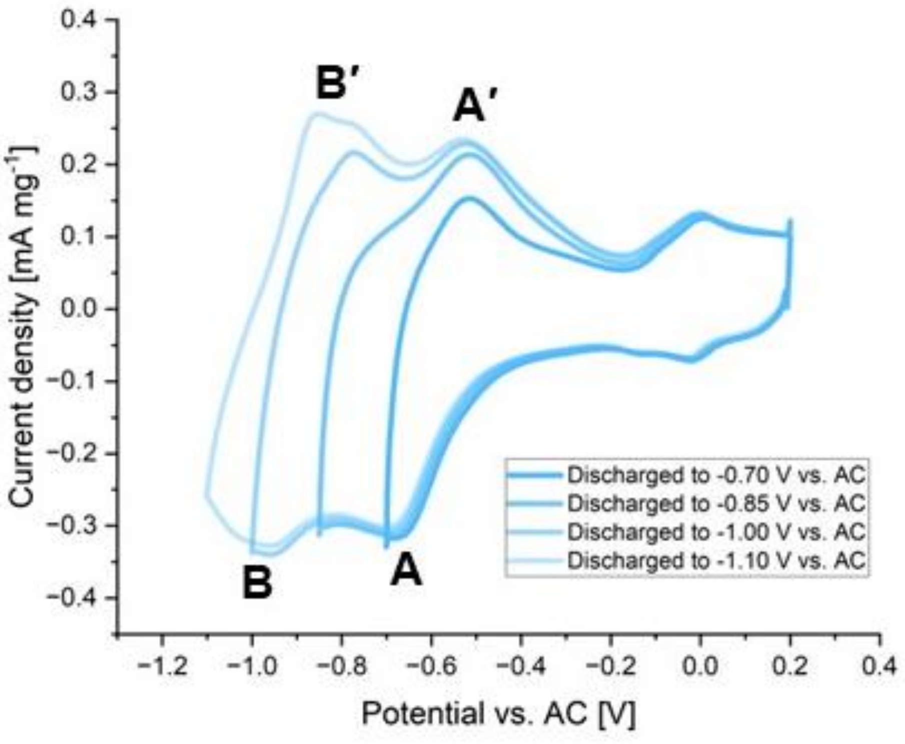

Figure S4. CV measurements of HVO-aqueous vs. AC at a sweep rate of 0.2 mV·s^{-1} in the potential range of -0.7 – 0.2 V (dark), -0.85 – 0.2 V, -1.0 – 0.2 V and -1.1 – 0.2 V vs. AC (light). The reduction peaks are labeled A and B, the associated oxidation peaks are labeled A’ and B’.

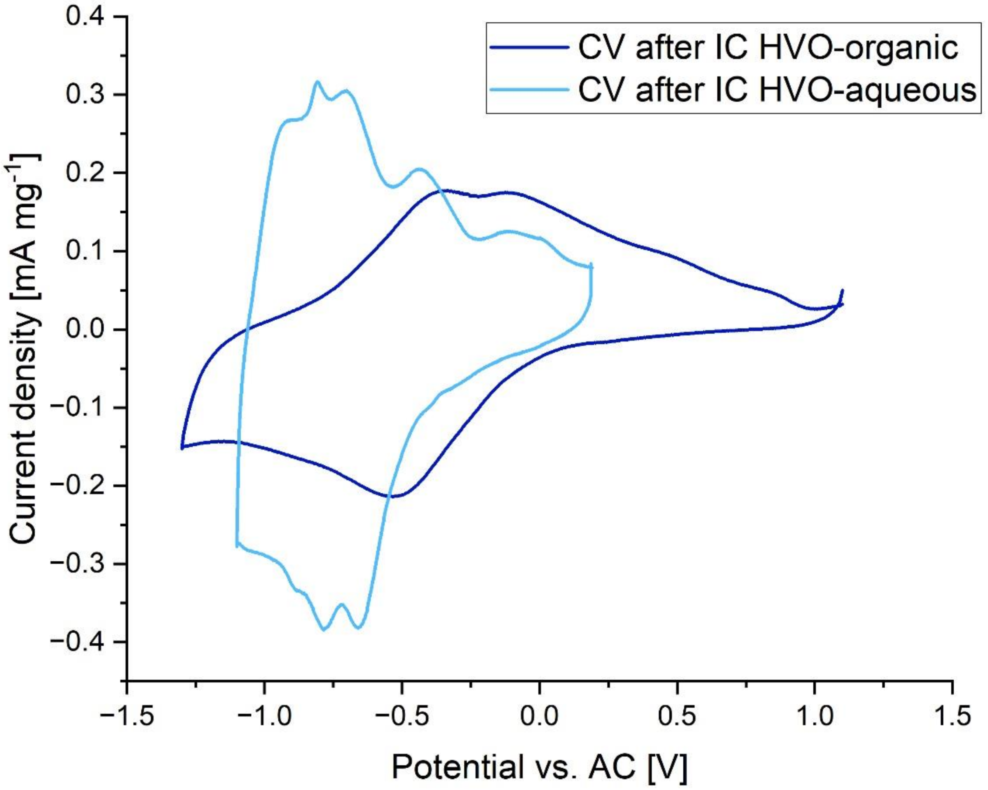

Figure S5. Comparison of CV measurements after the initial cycle (IC) of HVO-aqueous vs. AC (light-blue) and HVO-organic (dark-blue) at a sweep rate of 0.2 $mV \cdot s^{-1}$ in the potential range of -1.1 – 0.2 V vs. AC for HVO-aqueous and -1.3 – 1.1 V vs. AC for HVO-organic. The CV of HVO-organic is taken from Ref. [3]

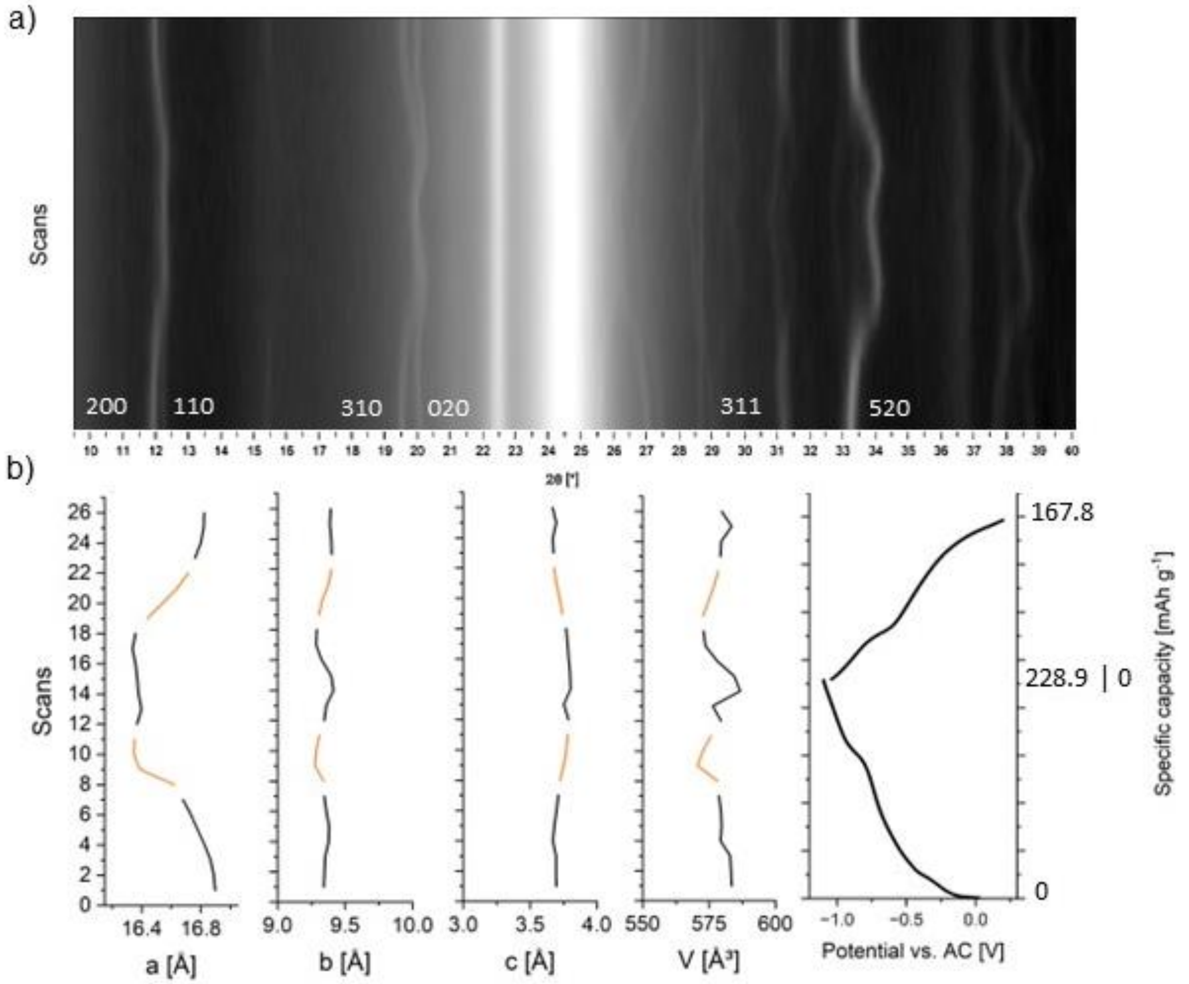

Figure S6. O*perando* XRD measurement during the galvanostatic initial cycle (IC) of HVO-aqueous at a current density of 80 mA·g^{-1} between -1.1 V and 0.2 V vs. AC. a) 2D diagram of *operando* XRD pattern (logarithmic intensities); b) refined lattice parameters of intercalated Mg^{2+} and Na^{+} HVO-aqueous.

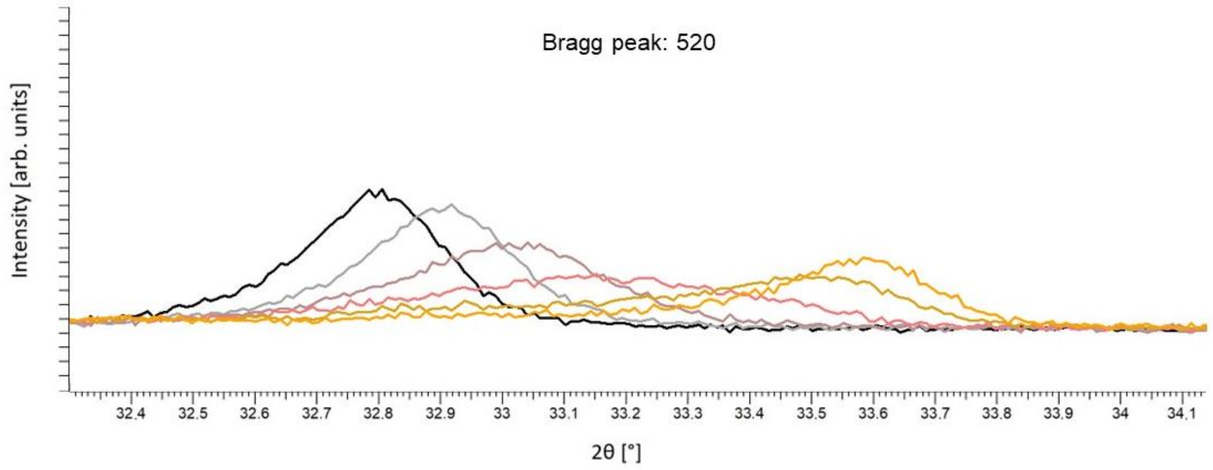

Figure S7. Detail view of the Bragg peak 520 during the transition from the solid solution process to the two-phase process and again to the solid solution process (scans 1 [black], 4 [grey], 5 [light-brown], 6 [pink], 7 [golden], and 8 [orange]) of HVO-aqueous vs. AC after the IC.

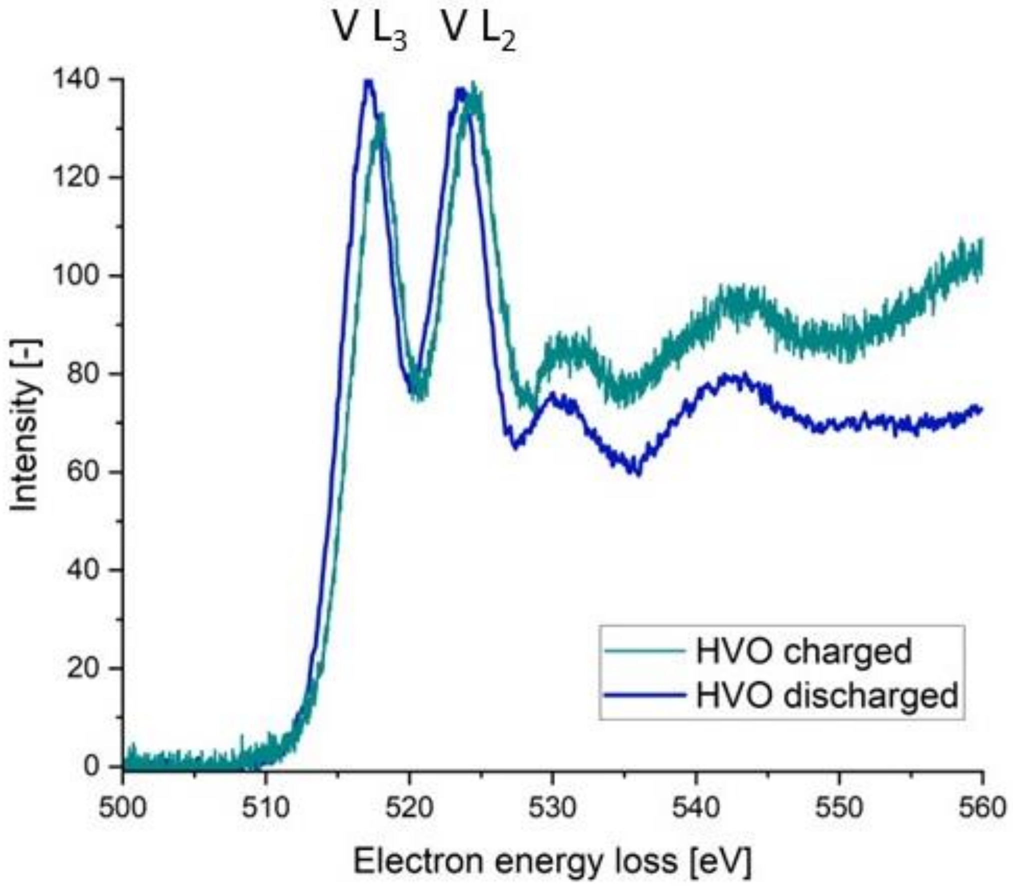

Figure S8. Post-mortem EELS of HVO after the initial galvanostatic discharge process (HVO discharged, blue) and after the initial galvanostatic charge process (HVO charged, turquois).

For EELS-measurements, an acceleration voltage of 60 kV was used. TEM grids were cleaned gently in He plasma (Zepto, Electronic Diener). All other characteristics and pre-steps (e.g. preparation of HVO samples) are described in the manuscript.

Table S3. Maximal changes (maximum – minimum values) of the lattice parameter and the unit cell volume of various ions in HVO in [%] referenced to the HVO unit cell volume at OCV. The values for Mg^{2+} (water contaminated organic electrolyte) and Na^{+} (organic electrolyte) are taken from Ref. [4]

Insertion ion	Mg^{2+} and Na^{+}	Mg^{2+}	Na^{+}
Electrolyte	aqueous	organic with water	organic
Δa [%]	3.3	3.8	2.5
Δb [%]	2.2	3.7	3.5
Δc [%]	4.5	2.8	n.a.
ΔV [%]	3.4	3.2	0.9

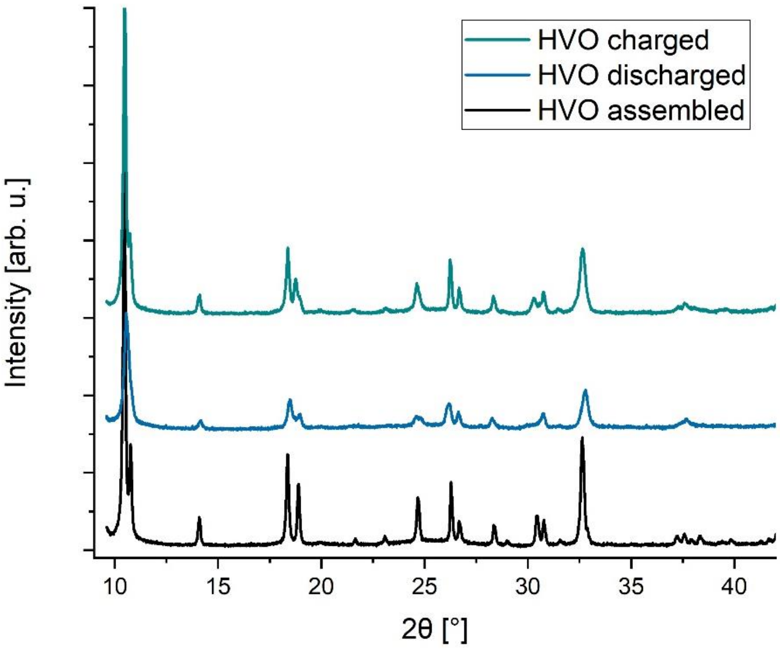

Figure S9. *Ex-situ* XRD measurements of HVO without cycling (HVO assembled; black), after the initial galvanostatic discharge process (HVO discharged, blue) and after the initial galvanostatic charge process (HVO charged, turquois).

All *post-mortem* XRD measurements were carried out immediately after finishing the electrochemical measurements. HVO assembled and HVO charged show reflexes at the same positions, whereas HVO discharged shows shifts and sometimes less distinct Bragg peaks (e.g. 320, 311, 520).

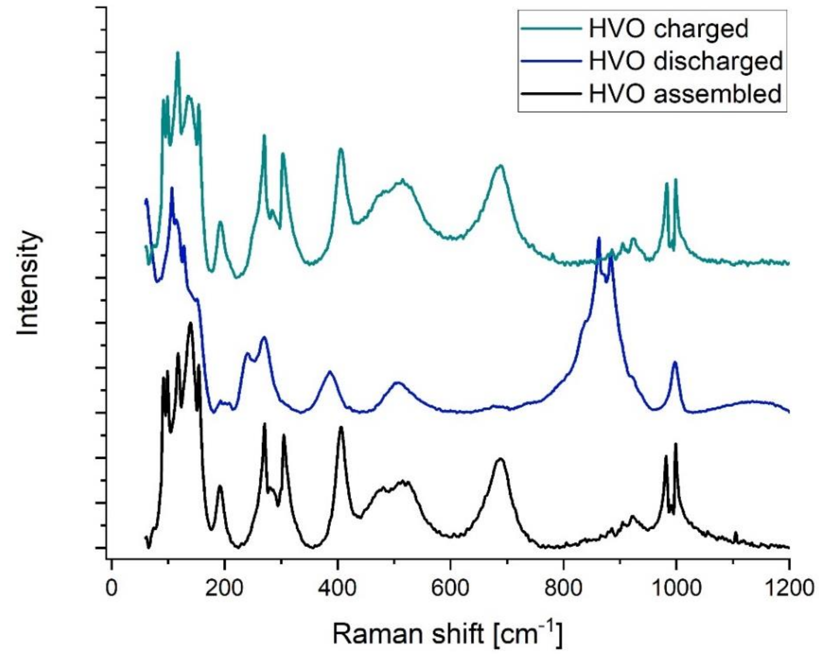

Figure S10. *Post-mortem* Raman spectra of HVO without cycling (HVO assembled; black), after the initial galvanostatic discharge process (HVO discharged, blue) and after the initial galvanostatic charge process (HVO charged, turquois).

All Raman acquisitions were carried out immediately after the electrochemical measurements finished. A laser with a wavelength of 532 nm was used and the power was set to 10 mW. The exposure time was set to 15 s and repeated 10 times.

Seperator after galvanostatic discharging HVO vs. AC:

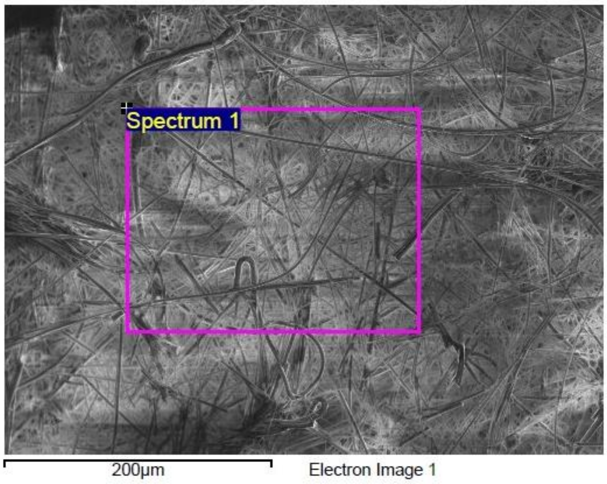

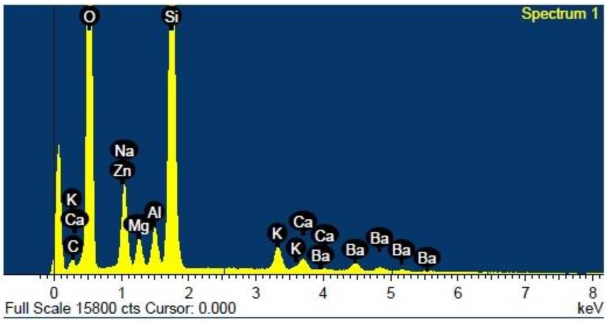

Element	Weight%	Atomic%
C K	5.61	9.20
O K	52.25	64.27
Na K	5.51	4.71
Mg K	1.87	1.52
Al K	2.24	1.63
Si K	22.08	15.47
K K	2.51	1.26
Ca K	1.30	0.64
Zn L	2.22	0.67
Ba L	4.41	0.63
Totals	100.00	

Seperator after galvanostatic charging HVO vs. AC:

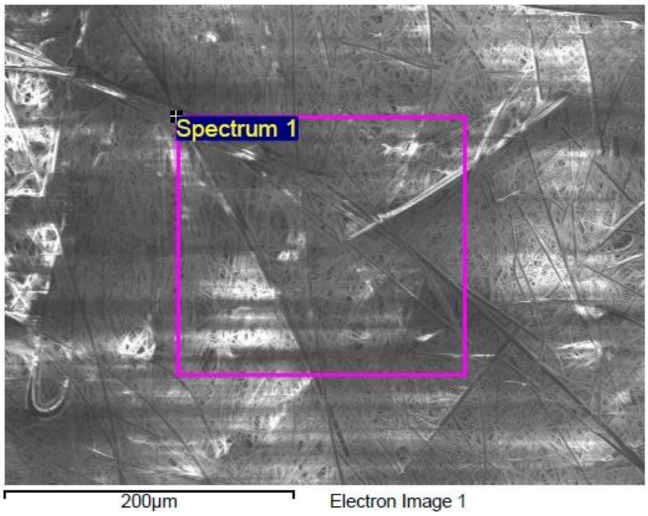

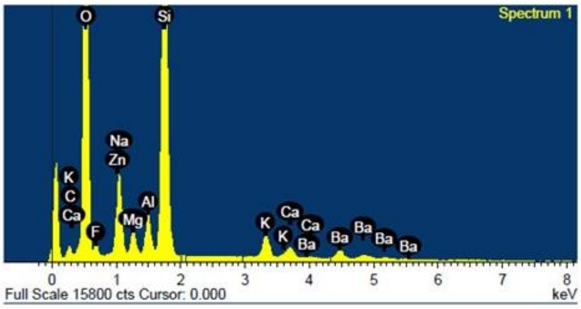

Element	Weight%	Atomic%
C K	5.15	8.54
O K	49.56	61.67
F K	2.84	2.98
Na K	5.46	4.73
Mg K	1.52	1.25
Al K	2.23	1.64
Si K	22.42	15.89
K K	2.48	1.27
Ca K	1.26	0.63
Zn L	2.36	0.72
Ba L	4.71	0.68
Totals	100.00	

Figure S11. *Post-mortem* EDX-measurements of the seperator after discharging/charging the cell using HVO vs. AC in a 0.5 M $Mg(NO_3)_2$ aqueous electrolyte.

The EDX-measurements confirm that no V was detected after the galvanostatic discharge or charge process, suggesting that no V is dissolved during the galvanostatic cycling process.

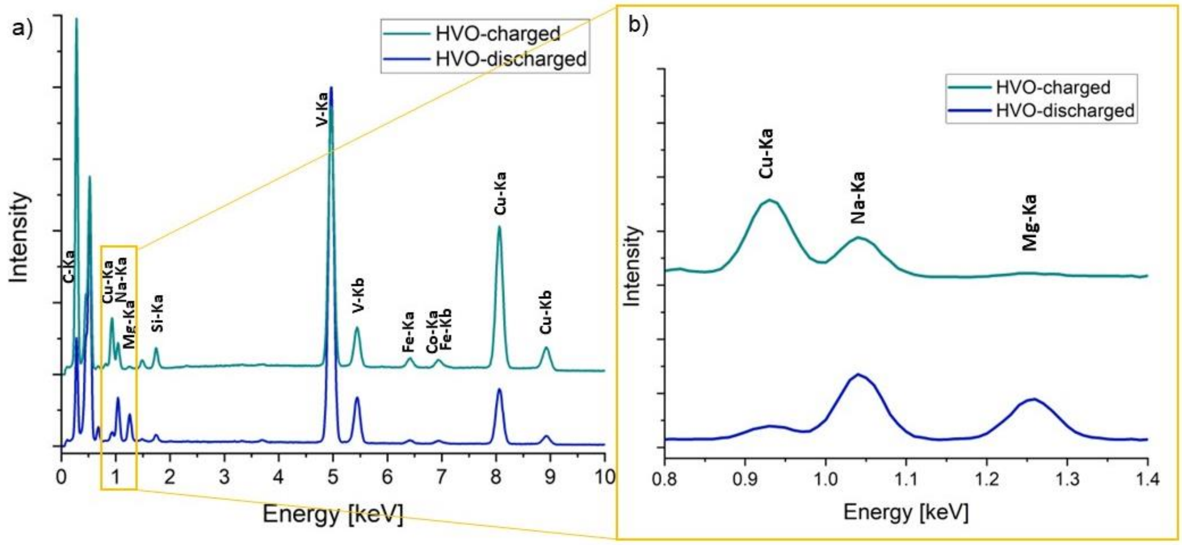

Figure S12. *Post-mortem* STEM/EDX spectra of HVO after the initial galvanostatic discharge process (HVO discharged, blue) and after the initial galvanostatic charge process (HVO charged, turquois). The Na-Ka and Mg-Ka intensities are visible for the discharge state and are decreased/absent for the charge state proving Na^+ and Mg^{2+} (de-)intercalation into the HVO structure. The Cu signal comes from the TEM grid. a) spectra from 0-10 keV b) spectra from 0.8-1.4 keV.

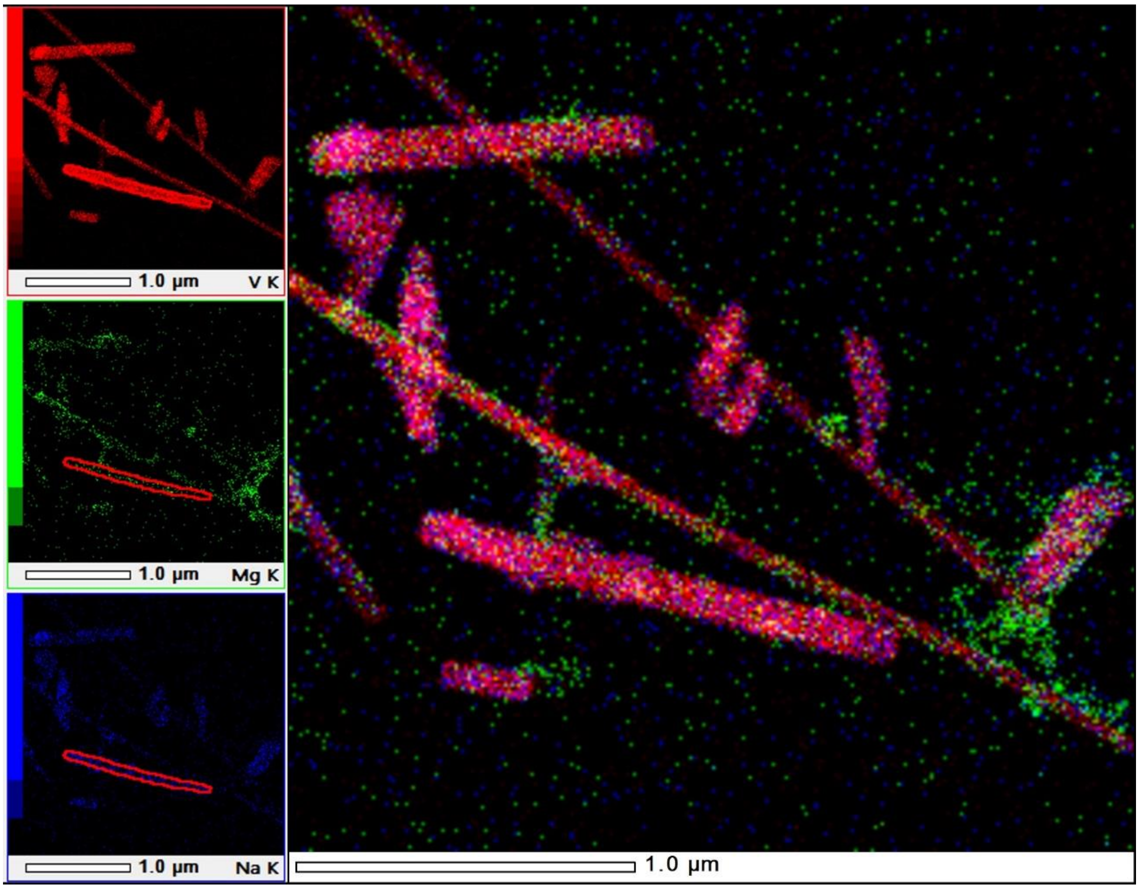

Figure S13. STEM image and EDX intensity maps of post-mortem HVO-aqueous nanofibers for the elements V:Mg:Na (scale bar: 1 µm). The area used for compositional analysis by EDX is colored in red. The results are displayed in the table below.

**Element**	**(keV)**	**Atom%**
**Na K**	1.041	0.57
**Mg K**	1.253	3.96
**V K**	4.949	95.46
**Total**		100.00

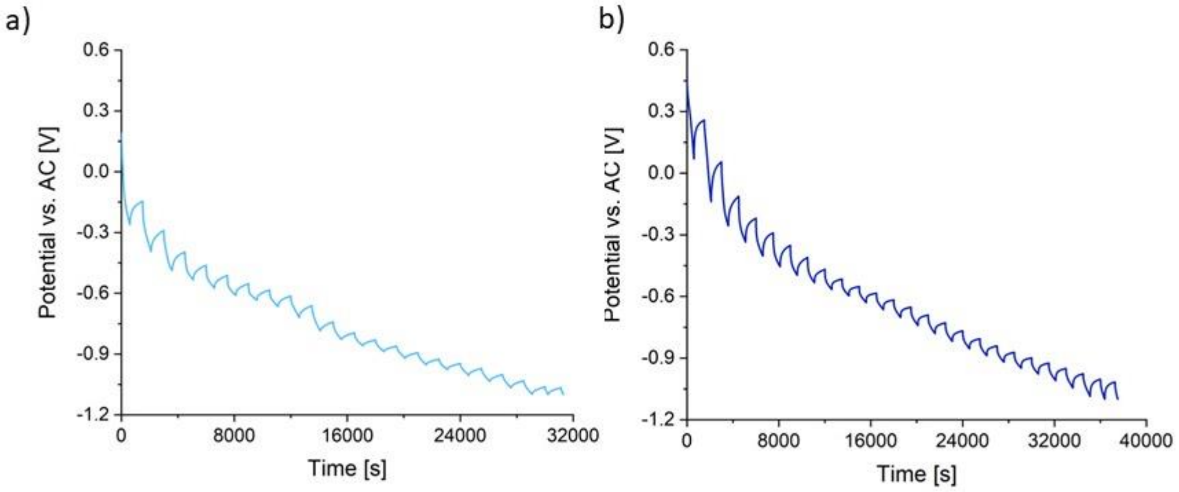

Figure S14. GITT potential feedback curves as a function of time during the initial discharge cycle at a current density of 100 mA·g^{-1} for a) of HVO-aqueous vs. AC b) HVO-organic vs. AC.

$$D = \frac{4}{\pi\tau}\left(\frac{m_B V_M}{S\,M_B}\right)^2\left(\frac{\Delta E_S}{\Delta E_t}\right)^2$$

m_B denotes the mass, V_M = 87 cm^3· mol^{-1} is the molar volume and M_B = 283 g· mol^{-1} is the molar mass of HVO. S is the area of the sample-electrolyte interface (SSA of HVO = 15.86 m^2· g^{-1}). ΔE_S is the steady-state cell voltage change for one step and ΔE_t is the total transient voltage change of the galvanic cell for an applied galvanostatic current for the time $\tau = 600$ s.

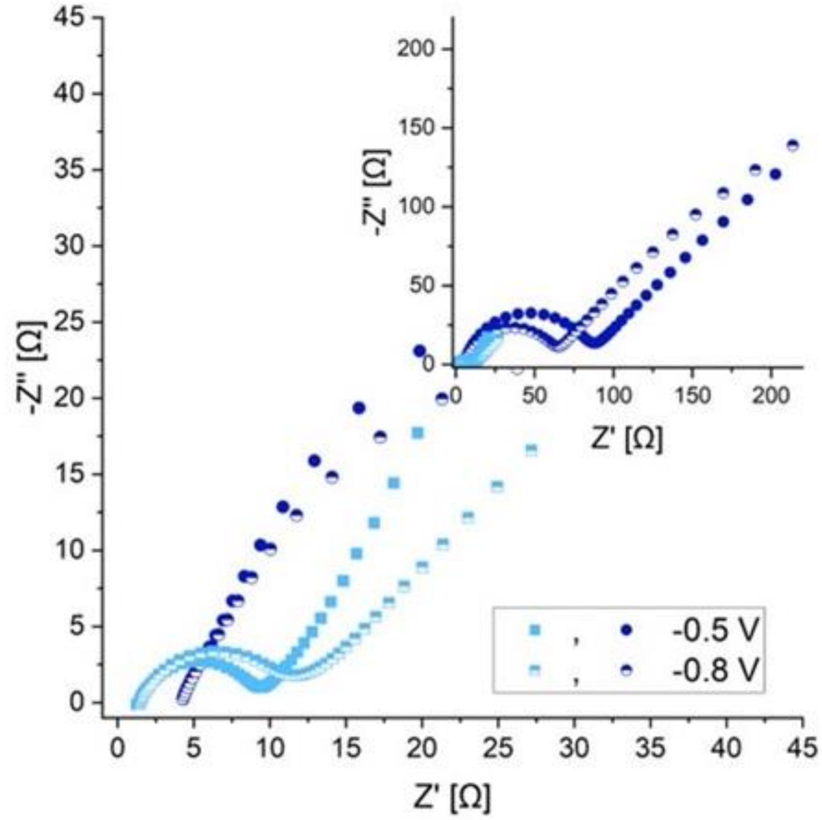

Figure S15. Nyquist plots of HVO-aqueous (light-blue) and HVO-organic (dark-blue) obtained from EIS measurements at potentials of -0.5 V and -0.8 V vs. AC after having completed the IC, a first CV and five discharge and charge cycles at a current density of 100 mA·g^{-1} and a sweep rate at 0.2 mV· s^{-1} in the potential range of -1.1 to 0.2 V vs. AC.

References:

[1] Zhan et al., *J. Alloys Compd*. **2009**, 479, 652-656.
[2] Söllinger et al., *ChemSusChem* **2021**, 14, 1112-1121.
[3] Söllinger *et al*., *Electrochim. Acta,* **2022***, 434,* 141294
[4] Söllinger *et al.*, *J. Phys. Chem. C*, **2024**, *128*, 5, 2255–2265
